# Dynamic brain-to-brain concordance and behavioral mirroring as a mechanism of the patient-clinician interaction

**DOI:** 10.1126/sciadv.abc1304

**Published:** 2020-10-21

**Authors:** Dan-Mikael Ellingsen, Kylie Isenburg, Changjin Jung, Jeungchan Lee, Jessica Gerber, Ishtiaq Mawla, Roberta Sclocco, Karin B. Jensen, Robert R. Edwards, John M. Kelley, Irving Kirsch, Ted J. Kaptchuk, Vitaly Napadow

**Affiliations:** 1 Department of Psychology, University of Oslo, Oslo, Norway.; 2 Norwegian Centre for Mental Disorders Research (NORMENT), Division of Mental Health and Addiction, Oslo University Hospital, Oslo, Norway.; 3 Athinoula A. Martinos Center for Biomedical Imaging, Massachusetts General Hospital, Charlestown, MA, USA.; 4 KM Fundamental Research Division, Korea Institute of Oriental Medicine, Daejeon, The Republic of Korea.; 5 Department of Radiology, Logan University, Chesterfield, MO, USA.; 6 Department of Clinical Neuroscience, Karolinska Institute, Stockholm, Sweden.; 7 Department of Anesthesiology, Brigham and Women’s Hospital, Boston, MA, USA.; 8 Endicott College, Beverly, MA, USA.; 9 Program in Placebo Studies and Therapeutic Encounter (PiPS), Harvard Medical School, Boston, MA, USA.

## Abstract

The patient-clinician interaction can powerfully shape treatment outcomes such as pain but is often considered an intangible “art of medicine” and has largely eluded scientific inquiry. Although brain correlates of social processes such as empathy and theory of mind have been studied using single-subject designs, specific behavioral and neural mechanisms underpinning the patient-clinician interaction are unknown. Using a two-person interactive design, we simultaneously recorded functional magnetic resonance imaging (hyperscanning) in patient-clinician dyads, who interacted via live video, while clinicians treated evoked pain in patients with chronic pain. Our results show that patient analgesia is mediated by patient-clinician nonverbal behavioral mirroring and brain-to-brain concordance in circuitry implicated in theory of mind and social mirroring. Dyad-based analyses showed extensive dynamic coupling of these brain nodes with the partners’ brain activity, yet only in dyads with pre-established clinical rapport. These findings introduce a putatively key brain-behavioral mechanism for therapeutic alliance and psychosocial analgesia.

## Teaser

Brain activity concordance between patients and clinicians supports therapeutic alliance and treatment relief of pain.

## INTRODUCTION

The patient-clinician interaction is fundamental to clinical care. Positive clinical encounters are associated with higher patient satisfaction, mutual trust (*1*), treatment adherence (*2*), and even clinical outcomes (*3*–*5*). Conversely, suboptimal interactions may propagate miscommunication (*6*), clinician burnout (*7*), patient distrust (*8*), and discourage care seeking (*9*). The patient-clinician relationship is also likely to account for a substantial part of psychologically mediated relief (e.g., placebo analgesia) (*10*). However, clinical engagement is often considered an intangible “art of medicine,” and scientific inquiry into the specific underpinning mechanisms has been minimal. A scientific understanding of the neurobiological and behavioral mechanisms supporting the patient-clinician interaction may be key to harnessing this untapped potential to improve clinical care.

A number of neuroimaging studies have established that brain regions including the temporoparietal junction (TPJ), anterior insula (aINS), and ventrolateral prefrontal cortices (vlPFCs) are implicated in social processes such as empathy and theory of mind (inferring the mental state of others) (*11*), which may also be relevant for the clinical encounter. A recent study indicated that this brain circuitry is activated in clinicians applying pain treatment to individuals appearing to be in pain (*12*). However, while most neuroimaging studies have used single-subject experimental designs, it is increasingly recognized that understanding the complex neural dynamics of social interactions, such as in the clinical dyad, requires the investigation of simultaneous brain activity in patients and clinicians during actual interaction (*13*).

For example, a large literature points to behavioral mirroring and physiological concordance as fundamental to human affiliation and bonding (*14*, *15*). In the context of clinical interaction, verbal (*16*) and nonverbal (*17*) behavioral synchrony between patients and clinicians is associated with better therapeutic effectiveness and relationship quality (*18*). Furthermore, concordance in sympathetic nervous system activation has been associated with higher physician empathy and less emotional distance (*19*). Recent functional brain imaging studies of two (or more) people during interaction (i.e., hyperscanning) have found that activity in social mirror networks synchronizes between individuals when socially interacting (*20*, *21*), and stronger coupling may reflect more successful communication (*22*), suggesting that concordance of brain activity in social mirroring networks may play a key role in social interaction (*13*, *23*).

**Fig. 1:**
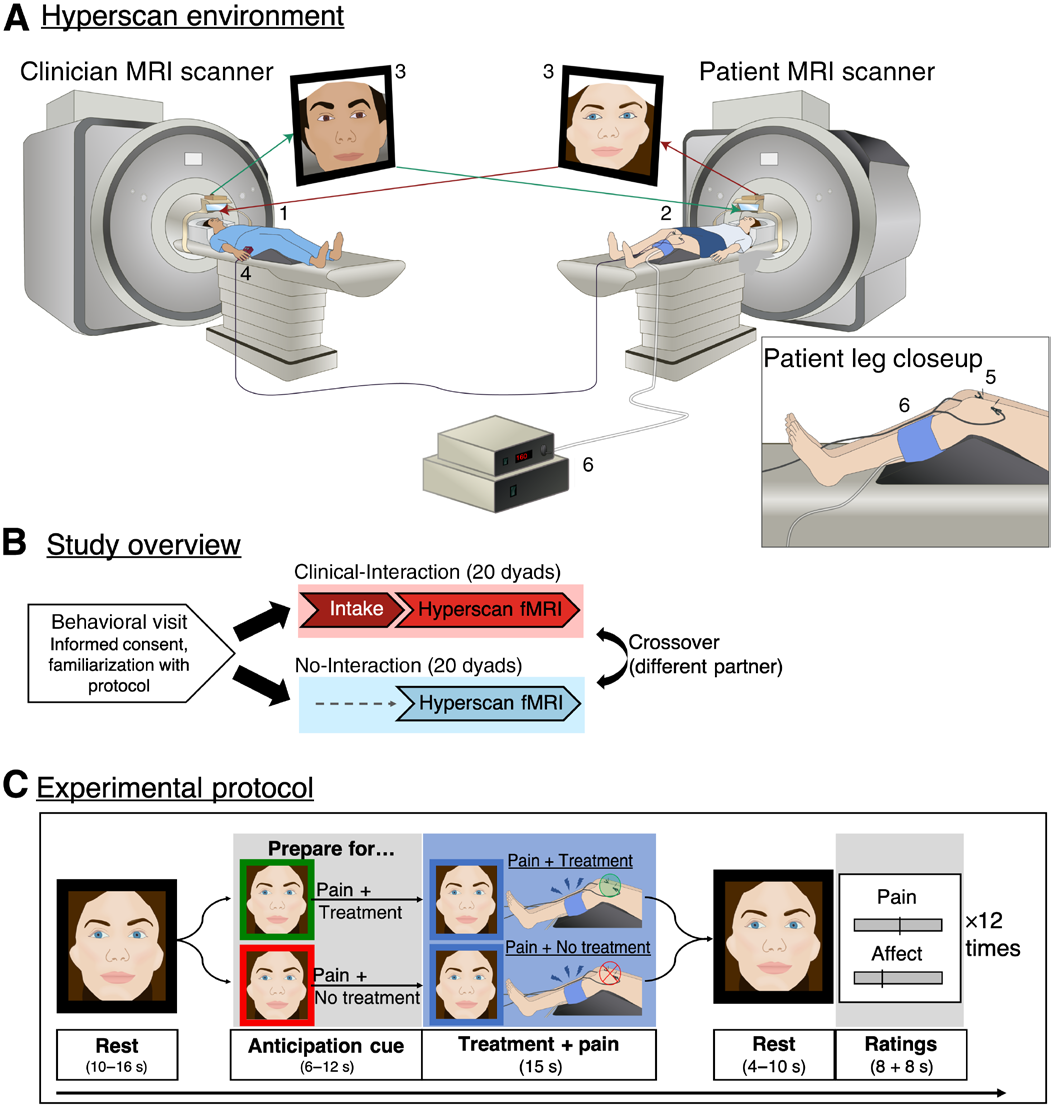
Study setup. (**A**) The fMRI hyperscanning environment. The clinician (1) and patient (2) were positioned in two different 3T MRI scanners. An audio-video link enabled online communication between the two scanners (3), and video images were used to extract frame-by-frame facial expression metrics. During simultaneous acquisition of blood oxygen level–dependent (BOLD)–fMRI data, the clinician used a button box (4) to apply electroacupuncture (EA) treatment (real/sham, double-blind) to the patient (5) to alleviate evoked pressure pain to the leg (6; Hokanson cuff inflation). Pain and affect related to the treatment were rated after each trial. (**B**) Study overview. After an initial behavioral visit, each individual participated in a Clinical-Interaction (hyperscan preceded by a clinical intake) and No-Interaction condition (hyperscan without a preceding intake), in a counterbalanced order, with two different partners. (**C**) Experimental protocol. Each hyperscan was composed of 12 repeated trials (four verum EA, four sham EA, and four no treatment) in a pseudo-randomized order. After a resting period (far left), both participants were shown a visual cue to indicate whether the next pain stimulus would be treated (green frame) or not treated (red frame) by the clinician. These cues prompted clinicians prepare to either apply or not apply treatment while evoking corresponding anticipation for the patient. Following the anticipation cue, moderately painful pressure pain was applied to the patient’s left leg, while the clinician applied or did not apply treatment, respectively. After another resting period, participants rated pain (patients), vicarious pain (clinicians), and affect (both) using a visual analog scale (VAS).

Here, we investigated patient-clinician mirroring in facial expressions and dynamic brain activity concordance as potential mechanisms supporting clinical outcomes mediated by patient-clinician interactions. We used functional magnetic resonance imaging (fMRI) to record brain activity simultaneously (fMRI hyperscanning) in patients with chronic pain and clinicians (acupuncturists) during an ecologically valid yet experimentally controlled clinical encounter, in which the clinician treated the patient to reduce evoked pain (Fig. 1A).

We enrolled 45 participants, including 23 female patients with chronic pain diagnosed with fibromyalgia (FM) for at least 1 year and 22 acupuncture clinicians (15 female). Each participant was matched with up to two partners, forming a total of 40 distinct, interacting dyads. Each dyad was scanned under one of two conditions (counterbalanced order): Under the “Clinical-Interaction” condition, the clinician performed a clinical consultation and intake with the patient before MRI scanning to enable the dyad to establish clinical rapport. The “No-Interaction” control condition was identical to Clinical-Interaction except that the patient and the clinician had not had an intake and were only introduced briefly before scanning (Fig. 1B). We hypothesized that dynamic brain activity concordance would be enhanced for Clinical-Interaction, in which the patient and clinician had a preestablished social relationship, relative to the No-Interaction control, in which no such relationship had been established. Because of data loss, we obtained complete MRI data from 37 dyads. See Methods for comprehensive methodological details.

## RESULTS

### Therapeutic alliance

Each participant completed four sessions: (i) a behavioral session for informed consent and familiarization with protocol; (ii) a clinical intake session, in which the clinician (acupuncturist) performed an intake with the patient, encouraged to be “as similar as possible to your daily practice” to maximize ecological validity; (iii) a Clinical-Interaction MRI on a separate day after the intake, in which the same patient and clinician were scanned together during a pain treatment session; and (iv) a No-Interaction MRI to control for the social relationship established at the intake. The order of Clinical-Interaction MRI and No-Interaction MRI was counterbalanced between participants (Fig. 1B). Different MRI visits were always on separate days.

The Consultation and Relational Empathy (CARE) (*24*) scale was collected after each session as a proxy for therapeutic alliance. A repeated-measure analysis of variance (ANOVA) confirmed that patients reported different levels of therapeutic alliance depending on the context of the dyadic clinical interaction [*F*(1.34,18.76) = 20.82, *P* < 0.001, η_p_^2^ = 0.60]. Planned direct comparisons indicated significantly lower CARE scores for No-Interaction MRI (mean ± SD = 32.19 ± 8.09), compared to Intake [mean ± SD = 42.20 ± 4.25, *t* = 5.84, *P* < 0.001, Cohen’s *d* = 1.46, 95% confidence interval (CI) = 5.39 to 14.61] and Clinical-Interaction MRI (mean ± SD = 41.63 ± 5.11, *t* = 5.21, *P* < 0.001, *d* = 1.30, 95% CI = 4.56 to 14.32) contexts (fig. S1). No significant difference in CARE scores was noted between Intake and Clinical-Interaction MRI (*t* = 0.63, *P* = 0.54, *d* = 0.16, 95% CI = −1.85 to 2.97) sessions. A similar pattern was seen for clinician-rated therapeutic alliance (see fig. S1). These results support the ability of the intake to robustly establish therapeutic alliance and clinical rapport, which was then carried over to the Clinical-Interaction MRI session.

### Evoked pressure pain, vicarious pain, and treatment-related affect

MRI-compatible video cameras allowed participants to communicate nonverbally (e.g., eye movement and facial expressions) throughout hyperscanning. During block-design fMRI, patients received 12 moderately painful cuff pressures to the left leg (Fig. 1C and see Supplementary Materials and Methods for details on stimulus presentation). Enrolling acupuncture practitioners as clinicians allowed for therapy to be administered during hyperscanning, using remote, but ecologically valid, controlled electroacupuncture (EA) through two needles placed above the patients’ knee (pseudo-randomized verum, sham, and overt No-Treatment, 15-s duration). Before each pain stimulus, both participants were given a visual cue (6- to 12-s jittered, frame around face changing color), indicating whether upcoming pain stimuli would be accompanied by Treatment (green) or No-Treatment (red). For patients, this cue elicited an anticipation of receiving or not receiving treatment for the upcoming pain, whereas for clinicians, this prompted them to prepare for whether to apply treatment. During cuff inflation, the clinician correspondingly pressed and held either the “Treatment” button or a different “No-Treatment control” button. After each stimulus (4- to 10-s jittered), the patients and clinicians rated pain intensity (patients), vicarious pain (clinicians), and affect (patients and clinicians) using visual analog scale (VAS).

**Fig. 2:**
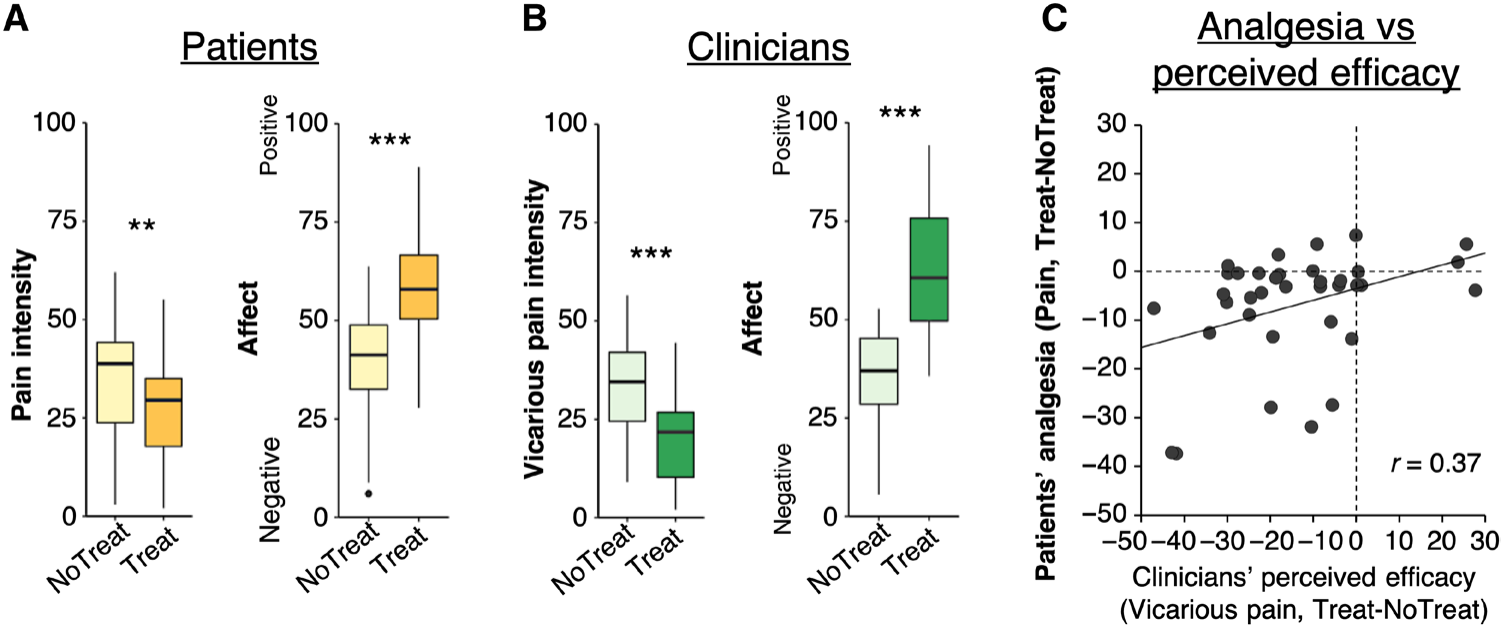
Self-reported pain and affect during fMRI hyperscanning. (**A**) Patients reported less pressure pain intensity when being treated by the clinician, relative to no-treatment trials. Furthermore, they reported feeling more positive affect while being treated, relative to no-treatment trials [VAS rating, “How did you feel about (not) getting treated with electroacupuncture?”; anchors, extremely negative/positive]. (**B**) Correspondingly, clinicians thought that patients had less pain during treatment trials relative to no-treatment trials, and they reported more positive affect while treating relative to not treating [VAS rating, “How did you feel about (not) doing the electroacupuncture?”; anchors, extremely positive/negative]. (**C**) A correlation between patients’ ∆Pain (Treat-NoTreat) and clinicians’ ∆Vicarious pain (Treat-NoTreat) difference scores suggested that for patients who reported greater pain relief, their clinician also perceived higher treatment efficacy. **P < 0.01 and ***P < 0.005.

There was no significant difference in pain between sham and verum EA (*t* = 0.83, *P* = 0.42), and an equivalence test [α = 0.05, equivalence bounds: Cohen’s d_z_ = −0.5 – 0.5 (10)] indicated that the difference between verum and sham EA was statistically equivalent to zero (*t* = 2.75, *P* = 0.005), supporting combining these collectively as treatment for subsequent analyses. Therefore, these conditions were pooled together as Treatment, collectively, for further analyses, and Treatment – No-Treatment differences are referred to as “analgesia” (see Supplementary Materials and Methods for details). For patients’ pain intensity, a repeated-measures ANOVA confirmed a main effect of “Treatment condition,” in which pain intensity was rated significantly lower for Treatment (mean ± SD = 26.32 ± 15.92), relative to No-Treatment [mean ± SD = 32.94 ± 17.98, *F*(1,15) = 9.79, *P* = 0.007, η_p_^2^ = 0.40, CI = 1.02 to 12.22; Fig. 2A]. There was no main effect of “Clinical context” [levels: Clinical-Interaction and No-Interaction, *F*(1,15) = 0.04, *P* = 0.84, η_p_^2^ = 0.003, 95% CI = −8.03 to 8.03] and no statistical interaction between Clinical context and Treatment condition [*F*(1,15) < 0.01, *P* = 0.98, η_p_^2^ < 0.01], suggesting that pain intensity and analgesia were comparable across different clinical interaction contexts. Furthermore, there were no interactions involving “Order” (*P*s > 0.12). However, an analysis of covariance (ANCOVA) confirmed that patient analgesia was significantly associated with subjective evaluations of the relationship, indicating that in dyads where relationship quality was rated more highly, patients reported stronger analgesia [hyperscan relationship scale score (“HRS score”), *F*(1,295) = 7.36, *P* = 0.007, η_p_^2^ = 0.02]. There were no significant main effects or interactions involving clinical interaction context (*P* > 0.06) and “HRS item” (*P* > 0.72), suggesting that the association between analgesia and relationship evaluation was comparable across HRS items and clinical interaction contexts.

For clinicians’ ratings of vicarious pain, there was a main effect of Treatment condition, in which vicarious pain was rated as significantly lower for Treatment (mean ± SD = 18.52 ± 13.62) relative to No-Treatment [mean ± SD = 33.06 ± 18.79, *F*(1,15) = 17.27, *P* < 0.001, η_p_^2^ = 0.55, 95% CI = 7.27 to 21.82; Fig. 2B]. There was no main effect of Clinical context [*F*(1,15) = 0.51, *P* = 0.49, η_p_^2^ = 0.04, CI = −7.19 to 14.70], no statistical interaction with treatment condition [*F*(1,15) = 0.51, *P* = 0.49, η_p_^2^ = 0.04] and no interactions involving Order (*P* > 0.61). Furthermore, patients’ analgesia (∆Pain, Treat-NoTreat) correlated with clinicians’ perceived treatment efficacy (∆Vicarious pain, Treat-NoTreat), such that for patients who reported greater pain relief, their clinician also perceived higher treatment efficacy (*r* = 0.37, *P* = 0.02), supporting patients’ ability to communicate their subjective pain to their clinician (Fig. 2C).

To investigate the relevance of this patient-clinician correspondence for therapeutic outcome, we investigated whether individual differences in clinicians’ ability to accurately estimate their patient’s pain was associated with patient analgesia. Specifically, for each dyad, we first calculated a correlation coefficient between the patient’s trial-by-trial pain ratings and the clinician’s vicarious pain ratings. These values, serving as a proxy for clinician accuracy in evaluating the patient’s pain, were then *r*-to-*z* transformed and correlated with patient analgesia scores. This analysis indicated that stronger patient analgesia was associated with higher patient-clinician correspondence in pain/vicarious pain (*r* = −0.39, *P* = 0.017; fig. S2).

Correspondingly, repeated-measures ANOVAs on ratings of affect indicated that both patients [*F*(1,15) = 10.69, *P* = 0.005, η_p_^2^ = 0.416, 95% CI = 8.37 to 29.81] and clinicians [*F*(1,15) = 12.35, *P* = 0.003, η_p_^2^ = 0.47, 95% CI = 13.18 to 39.78] felt more positively about Treatment trials than No-Treatment trials (Fig. 2, A and B), while ratings were comparable across Clinical-Interaction and No-Interaction contexts [patients: *F*(1,15) = 0.02, *P* = 0.90, η_p_^2^ = 0.001, 95% CI = −5.25 to 2.11; clinicians: *F*(1,15) = 0.01, *P* = 0.92, η_p_^2^ < 0.01, 95% CI = −6.48 to 5.99]. There were no Clinical Context * Treatment statistical interactions [patients: *F*(1,15) = 0.57, *P* = 0.46, η_p_^2^ = 0.001; clinicians: *F*(1,15) = 1.3, *P* = 0.27, η_p_^2^ = 0.09], indicating that affect was comparable across scans. There were no significant statistical interactions involving order (patients: *P* > 0.26; clinicians: *P* > 0.11).

### Facial mirroring was associated with placebo analgesia and therapeutic alliance

**Fig. 3:**
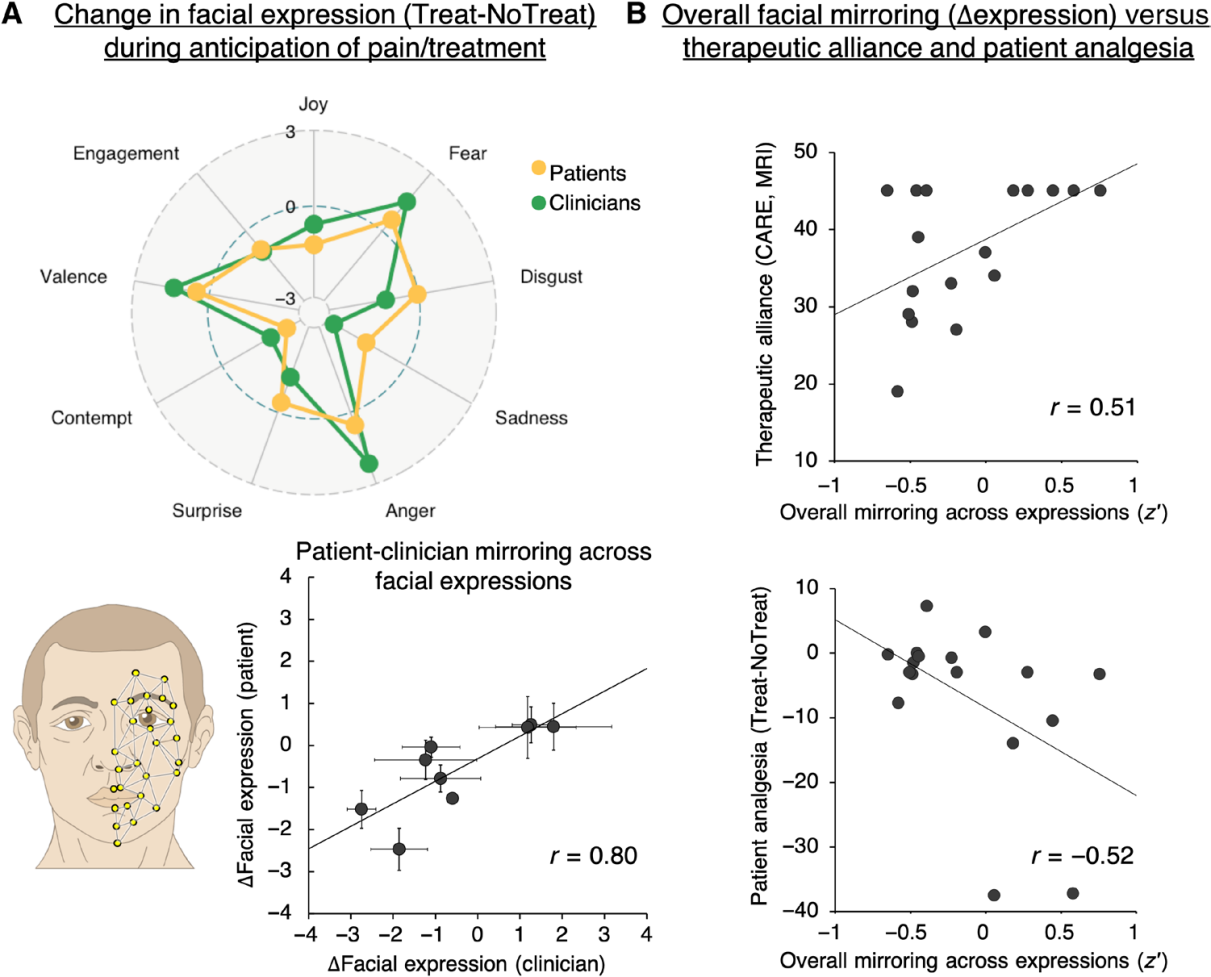
Patient-clinician mirroring in facial expressions during the therapeutic encounter. We used automated detection of facial muscle units, which were used to calculate frame-by-frame emotional expression scores (Affectiva, Cambridge, MA). (**A**) For the whole group, we found strong correspondence in treatment-induced change (Treat-NoTreat) between patients and clinicians across expressions. (**B**) To assess facial mirroring across expressions, we calculated the Treatment-induced (Treat-NoTreat) change for each expression for the patient and clinician, and subsequently Pearson’s coefficients (*r*-to-*z* transformed), across expressions within each dyad. This approach enabled higher sensitivity to differences in patterns of expressions between different dyads, compared to, e.g., assessing similarity within single expression metrics. We found that increased facial mirroring (overall, across all expressions) was associated with higher therapeutic alliance and stronger patient-reported analgesia (more negative values mean stronger pain reduction).

In-scanner videos were recorded and processed using automated facial feature (expression) extraction (Affectiva, Cambridge, MA). Because of data loss, a limited sample was used for analyses involving facial expressions (*n* = 17; see Methods for details). Average values for individual features were calculated for each trial. To assess treatment-related change in facial mirroring, we then calculated the correlation coefficient (*r*-to-*z* transformed) between patients and clinicians for the Treat-NoTreat change score across all features, resulting in one overall facial mirroring score per dyad (Fig. 3A). During anticipation of pain, facial mirroring across expressions correlated significantly with therapeutic alliance at MRI (*r* = 0.51, *P* = 0.036) and patients’ ratings of analgesia (*r* = −0.52, *P* = 0.031; Fig. 3B). There was a positive, yet nonsignificant, association between facial mirroring and right TPJ (rTPJ) concordance (*r* = 0.36, *P* = 0.15).

### Brain activation associated with pain and analgesia

To investigate treatment-related change in brain processing of evoked pressure pain, we first performed a whole-brain group general linear model (GLM) for all patients, for the contrast Treatment – “No-Treatment,” which indicated increased fMRI activation of bilateral vlPFC, TPJ, dorsolateral PFC (dlPFC), and medial PFC (mPFC), in addition to left superior temporal sulcus (STS) for treated, relative to nontreated, pain (fig. S3). We then investigated brain circuitry associated with individual treatment analgesia in the patients’ brain. A whole-brain regression analysis showed that stronger analgesia (NoTreat-Treat pain ratings) was associated with greater treatment-related increase in patients’ right vlPFC, precuneus, visual circuitry, and a cluster in the inferior parietal lobule (IPL)/supramarginal gyrus (SMG) during pain (Treat-NoTreat) (fig. S4).

### Brain activation associated with clinicians’ evaluation of patients’ pain

To investigate the clinician’s brain activation associated with evaluation of the patient’s pain during treatment provision, we performed a whole-brain regression analysis with each clinician’s ability to accurately evaluate the patient’s pain (patient-clinician correspondence in pain/vicarious pain ratings) as a regressor for clinicians’ brain activation during the Pain/Treatment period (Treat-NoTreat). The results indicated that increased treatment-related activation of the clinicians’ vlPFC, SMG/IPL, and STS was associated with higher patient-clinician correspondence in pain/vicarious pain intensity (fig. S5).

### Shared activation between patients and clinicians in brain circuitry associated with social mirroring

**Fig. 4:**
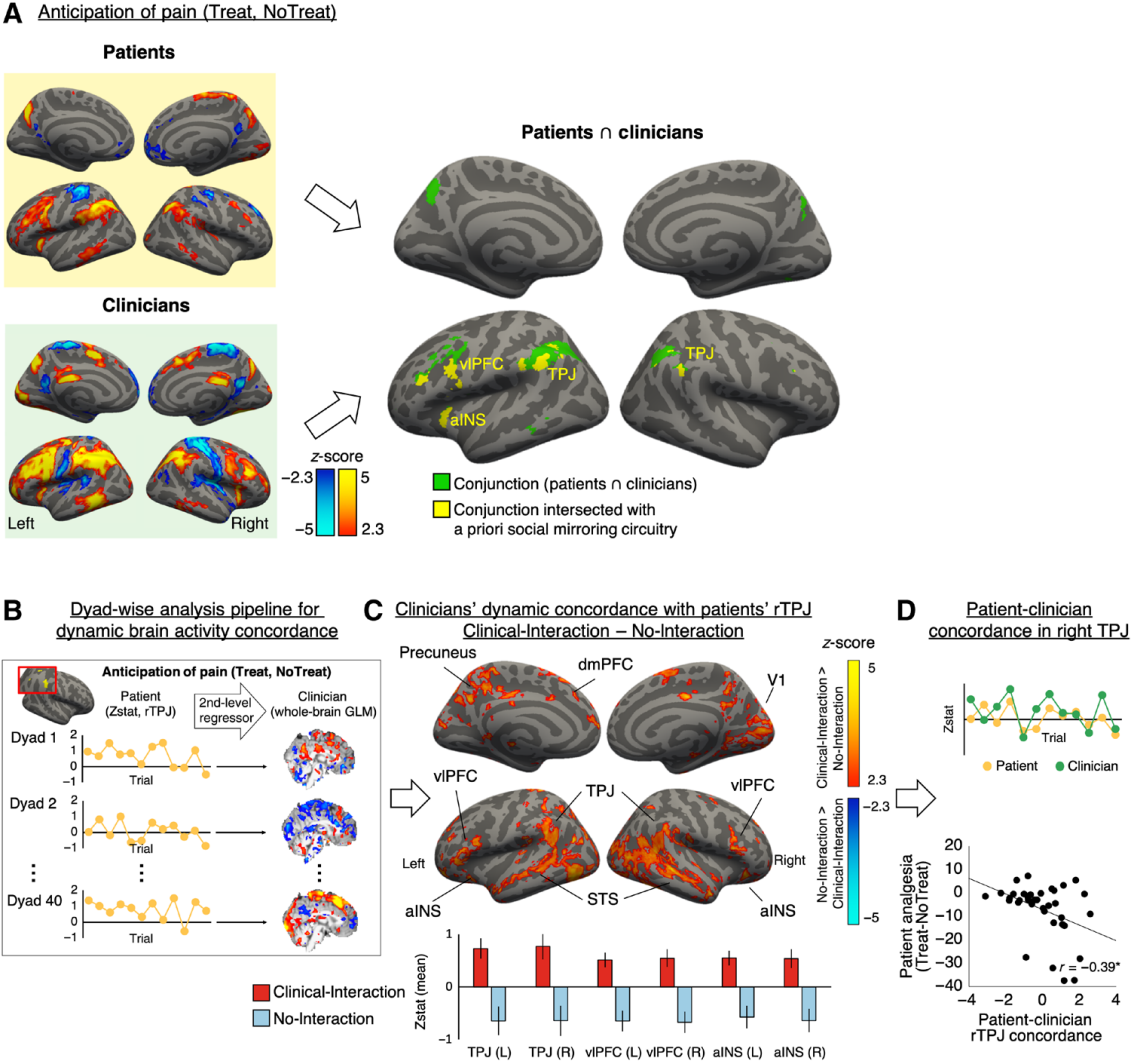
Shared brain activation and dynamic concordance between patients and clinicians. (**A**) The left panel shows fMRI responses to anticipation of receiving pain (patients) (top left) and preparing to provide/not provide treatment (clinicians) (bottom left). A conjunction analysis of these activation maps demonstrated common anticipatory activation for patients and clinicians in brain circuitry implicated in social mirroring, theory of mind, and empathy, such as left vlPFC, aINS, bilateral TPJ, and left STS, in addition to the precuneus, a cluster comprising bilateral supramarginal/angular gyrus, and superior parietal lobule. (**B**) To assess dynamic concordance in brain activity between patients and clinicians, throughout the pain/treatment scan, we extracted trial-by-trial *z*-scores from the patient’s rTPJ, which were then used as regressors in the clinician’s second-level GLM. This analysis used trial-by-trial whole-brain contrast parameter estimates for the pain/treatment anticipation block. (**C**) A group-level analysis of clinician dynamic concordance with patients’ rTPJ showed that Clinical-Interaction, relative to the No-Interaction control, enhanced rTPJ concordance to circuitry implicated in mentalizing, empathy, and social mirroring, e.g., TPJ, vlPFC, aINS, and STS, in addition to visual circuitry and precuneus, a key node of the default mode network. The bottom panel shows mean Zstat values from extracted ROIs, with error bars indicating SEM. (**D**) For the enhanced rTPJ-to-rTPJ contrast, dynamic fMRI response was driven by increased concordance between patients and clinicians following Clinical-Interaction (top) (example dyad). Greater patient-clinician rTPJ concordance was associated with stronger patient analgesia (bottom).

Next, we investigated dynamic brain activity concordance between patients and clinicians, focusing on the anticipation period, when the relationship may affect brain activity without competing neural processing of nociceptive afference (for patients) or motor activity for treatment delivery (for clinicians), as during the pain/treatment period. To assess brain activity concordance, we first calculated brain response to anticipation of pain, relative to rest, collapsed over Treat/NoTreat conditions (Fig. 4A, left), as concordance related to therapeutic alliance and pain outcomes could be driven by social interaction during the anticipation of both treated and nontreated pain. Next, we performed a whole-brain voxelwise conjunction analysis using the minimum statistic to investigate brain circuitry commonly activated for both patients and clinicians, which provided regions of interest (ROIs) for dynamic concordance analyses. This group conjunction analysis demonstrated shared anticipatory activations between patients and clinicians in bilateral circuitry implicated in social mirroring, theory of mind, and social cognition (e.g., bilateral TPJ, left vlPFC, and left aINS). See fig. S6 for analyses of patients’ and clinicians’ brain responses during the pain phase.

### Social interaction enhanced patient-clinician dynamic concordance in brain activity

For each dyad, we then extracted each individual’s mean ROI *Z* statistical (Zstat) value from each trial, which were used as a regressor in a second-level GLM for their dyadic partner’s fMRI data, providing a whole-brain map of dynamic concordance with the partner’s ROIs for each dyad (see Methods and fig. S7 for details). The same ROIs, as identified by the group conjunction analysis, were used for all dyads. Using a dynamic metric is important as concordance is best defined by shared deviations in brain response across dyad members (*25*). Following Clinical-Interaction, dynamic (trial-to-trial) rTPJ_Patients_ concordance was evident with clinicians’ brain response in circuitry implicated in social mirroring, theory of mind, and social cognition (e.g., bilateral TPJ, vlPFC, and aINS), in addition to visual and executive control circuitry, and significantly differed from the No-Interaction context for these regions (Fig. 4, B and C). ROI extraction from the clinicians’ whole-brain maps demonstrated that concordance between patients’ and clinicians’ rTPJ (but not other ROIs from above, *r*s = −0.11 to 0.17, *P* > 0.5) was significantly associated with patients’ analgesia (*r* = −0.39, *P* = 0.017; Fig. 4D). Analyses exploring effects of Clinical-Interaction on dynamic concordance with other nodes of the social mirroring circuitry are shown in fig. S8.

While the primary focus of this investigation was on anticipatory concordance, we also explored the role of rTPJ concordance during the pain stimulation phase. rTPJ concordance during pain was not associated with patient-rated analgesia (*r* = −0.13, *P* = 0.44). Instead, rTPJ concordance during the pain stimulation phase was correlated with clinicians’ affect (Treat-NoTreat, *r* = 0.41, *P* = 0.01) related to having provided treatment but not with patients’ affect ratings (Treat-NoTreat, *r* = 0.01, *P* = 0.95).

### Patients’ treatment-related brain response to pain mediated the effect of rTPJ concordance on analgesia

**Fig. 5:**
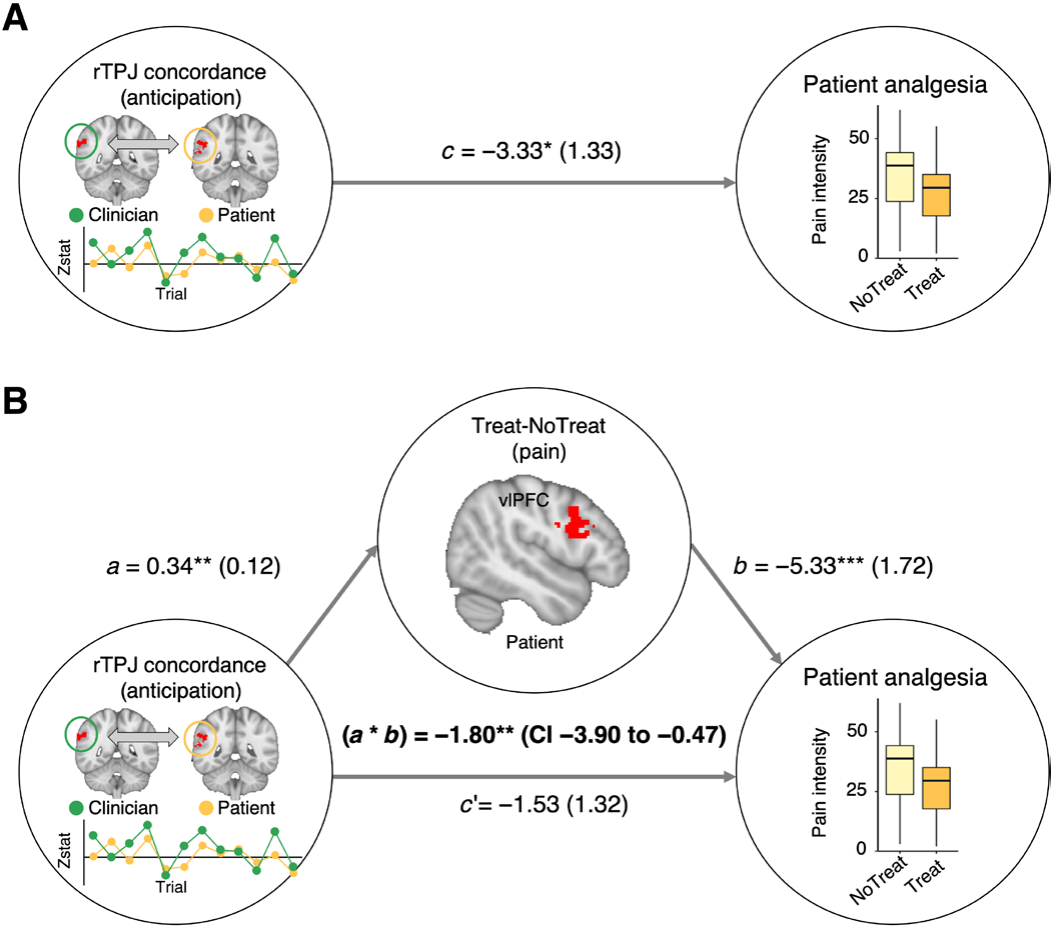
Patients’ treatment-related change in vlPFC mediated the association between rTPJ concordance and analgesia. (**A**) Anticipatory rTPJ concordance between patients and clinicians showed a direct linear association with patient analgesia. (**B**) A mediation analysis showed that Treatment-related change in patients’ vlPFC response during pressure pain statistically mediated the association between rTPJ concordance and patient analgesia, suggesting a mechanism in which patient-clinician rTPJ concordance recruits a pain modulatory vlPFC response in the patient’s brain. The brain metrics applied as dependent (rTPJ concordance) and mediator (vlPFC_Treat-NoTreat_) variables were derived from whole-brain analyses thresholded at *Z* = 2.3, *P* < 0.05, cluster-corrected for multiple comparisons. *P < 0.05, **P < 0.01, and ***P < 0.005.

Last, we explored whether concordance effects on analgesia were mediated by treatment-related change in specific social mirroring circuitry regions (e.g., vlPFC) for the patient during pain. We found that stronger treatment analgesia was associated with increased treatment-induced fMRI response in pain modulatory circuitry, e.g., vlPFC (fig. S4). The bootstrapped mediation analysis indicated a significant effect of the indirect path (*a* * *b* = −1.80, *P* = 0.006, 95% CI = −3.90 to −0.47), indicating that treatment-related change in patients’ vlPFC response during pain (Pain_Treat-NoTreat_) mediated the effect of anticipatory patient-clinician rTPJ concordance on analgesia (Fig. 5). Other nodes in the social mirroring circuitry activated during Pain_Treat-NoTreat_ did not significantly mediate this relationship (*P* > 0.07).

## DISCUSSION

We identified a putative brain-behavioral mechanism supporting the patient-clinician relationship and how it may influence clinical outcomes. We found that dynamic patient-clinician concordance in brain activity implicated in social mirroring and theory of mind was increased after the establishment of therapeutic alliance through a clinical interaction. Furthermore, stronger brain concordance was associated with stronger analgesia, an association that was mediated by activation of pain modulatory circuitry in the patient during pain. Finally, increased facial mirroring between patients and clinicians was associated with stronger therapeutic alliance and greater analgesia.

Patient-clinician behavioral synchrony and reciprocity are thought to support processes such as mutual empathy and therapeutic alliance (*26*) and thus constitute a cornerstone for patient-centered care (*18*, *27*). We found that circuitry implicated in social mirroring (TPJ, vlPFC, and aINS) was commonly activated in both patients and clinicians during anticipation of pain and treatment. Dyad-based analyses suggested that these nodes showed extensive dynamic coupling with the partners’ brain activity, but only in dyads who had established a clinical relationship before MRI (Clinical-Interaction), demonstrating that such dynamic brain-to-brain concordance was sensitive to modulation by the clinical relationship. Specifically, rTPJ-to-rTPJ concordance showed the strongest association with patients’ analgesia. The TPJ is a key hub in theory-of-mind processes, i.e., mentalizing about others’ thoughts and feelings (*28*). A recent meta-analysis of experimental fMRI studies on theory of mind and empathy found that TPJ was more strongly linked to mentalizing and moral cognition than to (emotional) empathy (*11*).

There were considerable differences between dyads in the correspondence between the patient’s pain intensity and the clinician’s estimation of the patient’s pain (vicarious pain), and the data indicated that for dyads where the clinician evaluated the patient’s pain more accurately, the patient showed stronger therapeutic pain relief. Furthermore, a fMRI regression analysis indicated that increased treatment-related activation of the clinician’s social mirroring circuitry (e.g., vlPFC, SMG/IPL, and STS), while providing treatment, relative to no treatment, was associated with higher clinician accuracy in evaluating the patient’s pain (i.e., greater ratings correspondence).

Our data further suggested a mechanism for how dynamic concordance during pain anticipation led to pain relief for the patient. During pain, patients reporting the strongest analgesia also showed the strongest treatment-induced activation in a number of regions including the vlPFC (fig. S4), which is implicated in both social mirroring (*29*) and psychosocially facilitated pain relief (*30*). We did not find that analgesia was associated with expectancy of treatment efficacy (patients’ expectations: mean ± SD = 4.00 ± 2.80, *r* = −0.12, *P* = 0.51; clinicians’ expectations: mean ± SD = 5.52 ± 2.53, *r* = −0.17, *P* = 0.34) nor with brain responses in other regions related to expectancy-induced pain modulation, such as pregenual anterior cingulate cortex (pgACC), dlPFC, and periaqueductal gray (*31*–*33*). Instead, stronger patient analgesia was associated with more positive evaluations of the social interaction (e.g., the patient’s feeling of comfort from seeing the clinician). This may reflect potential differences in the brain circuitry responsible for socially mediated, relative to expectancy-mediated pain relief. Our results suggest a putative mechanism for social context–induced pain relief by which patients’ treatment-related vlPFC activation during pain statistically mediates the effect of anticipatory rTPJ concordance on analgesia. While a large literature indicates a key role of expectations in placebo effects (*34*, *35*), a direct association between explicit expectation ratings and placebo effect magnitude has been highly variable in previous studies, especially for chronic pain (*5*, *36*, *37*). Furthermore, our approach primarily targeted the clinical relationship and therapeutic alliance and not the specific role of expectations. Thus, patient analgesia in this study may have been more strongly driven by these social interaction aspects, as hypothesized, and less by expectation-related mechanisms. Nevertheless, it is still possible that relevant expectations, overt or subliminal (*38*), may have changed dynamically over the course of the experiment and, thus, eluded our assessment.

While the impact of anticipatory rTPJ concordance on patient analgesia was mediated by treatment-related increase in the patient’s vlPFC activity during pain, rTPJ concordance during pain was not significantly associated with patient analgesia. During this pain/treatment period, there were important differences in task between patient and clinician (i.e., receiving pain stimulus versus button press to provide treatment, respectively). In contrast, the anticipation period was less confounded by differences in task, as there was a distinct lack of nociceptive input. Thus patient/clinician interaction, reflecting a relationship, could be evaluated during a less confounded state that still included a clinically relevant interaction. Instead, our results indicated that rTPJ concordance during this pain/treatment period was associated with clinicians’ affect related to having provided treatment, suggesting that the pain/treatment period was also characterized by therapy-linked rTPJ concordance. Future studies should more specifically disentangle the role for the social mirroring network and nociceptive processing regional concordance during pain stimulation, and in particular, how this concordance may differentially affect patients’ nociceptive (*39*) or higher-level pain modulatory processing (*40*).

A central question is why brain concordance and behavioral mirroring arises in the clinical encounter and is beneficial to patients. One possibility is that behavioral mirroring and synchrony may cause brain/physiological concordance, which, by promoting a positive affective-motivational state, leads to greater analgesia. From an evolutionary perspective, social affiliation signals support, care, and safety (*41*). One mode of this signaling may be behavioral synchronicity and neurobiological concordance, which are thought to support optimization of neural computation by reducing free energy and prediction errors (*42*) and, thus, represent a rewarding state associated with positive affect (*43*). The affective-motivational state induced by brain concordance may thus signal care and safety for the patient and reduce the perceived aversiveness/threat and, consequently, the intensity of the painful stimulus during the clinical context. This would be consistent with two influential theoretical frameworks for understanding pain as a symptom. First, the “motivation-decision model of pain” posits that the brain continually makes (unconscious) decisions about the importance of nociceptive signals giving rise to pain, depending on the context (*44*). Second, the “signaling theory of symptoms” posits that besides promoting self-protection, a main function of clinical symptoms such as pain is to motivate social signaling of the need for care (*45*). Once this need is met, these symptoms should be attenuated. Hence, a positive clinical context characterized by high rapport, therapeutic alliance, and biobehavioral concordance may serve as a safety signal for the patient, and consequently, the pain-evoking stimulus is deemed less salient, leading to analgesia. Recent studies have suggested behavioral mirroring and synchrony, e.g., in vocal acoustics (*16*), language style (*46*), posture (*17*), and gestures (*47*) as key features of clinical interactions. Here, we found that mirroring of facial expression was significantly associated with therapeutic alliance and analgesia.

Our study has several limitations. First, although we implemented a relatively naturalistic intake and consultation and strived to maximize ecological validity during testing, the MRI environment necessitates the omission of several often-important psychosocial aspects in real-life therapeutic interactions (e.g., touch and sensitive proximity) (*48*). Future studies may address these aspects via analyses of cortical concordance using electroencephalography or near-infrared spectroscopy hyperscanning, allowing dyads to be recorded while interacting verbally and nonverbally in the same room. Second, there may be important aspects of the clinical relationship that develop over time and cannot be captured after a single intake. While individual differences in analgesia were associated with social interaction quality, we did not find a mean group difference in analgesia between Clinical-Interaction and No-Interaction contexts. The data showed considerable variability across individuals in both relationship quality and pain outcomes during the MRI visit, both within the Clinical-Interaction and No-Interaction groups, which may have contributed to the inability to show a statistically significant group difference in pain outcomes. Future studies using a longitudinal design may better elucidate how brain concordance and therapeutic alliance develop over time and how this contributes to pain treatment outcomes.

In conclusion, our study used a comprehensive two-person approach to identifying a putative brain-behavioral mechanism of the patient-clinician interaction. The findings represent an important first step toward specifying the nonspecific components of the clinical encounter and to establish the neuroscience supporting the patient-clinician relationship.

## METHODS

### Participants

Licensed acupuncturists were recruited from the local community and had completed at minimum a 3-year masters-level program or were in their final year of training and interning in clinics [age: 44.32 ± 12.81 (mean ± SD); race: 18 Caucasian, 1 Hispanic, 1 African-American, 1 Asian, and 1 multiracial]. Patients with chronic pain diagnosed with FM for at least 1 year, meeting updated Wolfe and Häuser (*49*) criteria, were recruited for the “Patient” group [age: 39.95 ± 10.93 (mean ± SD); race: 18 Caucasian, 2 Hispanic, 2 African-American, and 1 multiracial, all female]. Clinicians ($150 per MRI session and $50 for non-MRI sessions) and patients ($100 per MRI session and $50 for non-MRI sessions) received monetary compensation for participation. The interval between the clinical intake and the Clinical-Interaction MRI was 8.32 ± 14.07 days. The order was counterbalanced (which was limited by difficult scheduling logistics; patients, 8 Clinical-Interaction first and 15 No-Interaction first; clinicians, 14 Clinical-Interaction first and 8 No-Interaction first], and each participant contributed to two dyads, paired with a different partner, to avoid carryover effects related to the relationship. Thus, each dyad was unique. We scanned a total of 40 dyads, whereby we obtained complete MRI data from 37 dyads (19 Clinical-Interaction and 18 No-Interaction, with 2 dyads incomplete because of scanner malfunction and 1 dyad incomplete because of patient withdrawal due to claustrophobia mid-scan). Furthermore, three patients (one ineligible and two due to scheduling issues) and two clinicians (due to scheduling issues) were enrolled but did not proceed to MRI scanning. Thus, 20 patients and 20 clinicians participated for at least one MRI visit and were included in dyad-based analyses. Of these, three patients (two due to scheduling issues and one due to claustrophobia) and three clinicians (two due to scheduling issues, one due to scanner discomfort) dropped out after completing one MRI visit. Thus, for paired analyses, 17 female patients with FM and 17 clinicians (12 female) completed both MRI visits. The Massachusetts General Hospital institutional review board approved the study, and all participants provided informed consent.

Since no relevant prior data existed on dynamic concordance, we could not estimate power using these dyad-based metrics. However, in our pilot data from clinicians providing treatment for the evoked pain of a patient confederate (*12*), we observed a within-subject average blood oxygen level–dependent (BOLD) percent change for treatment minus “control” (no treatment) of 1.25 ± 1.53 (mean ± SD). An a priori power analysis (paired, two-tailed, α = 0.05) indicated that a minimum of 15 participants (paired test) would be required for 85% power to detect this effect size (RStudio, function pwr.t.test, package pwr).

### Overall study protocol

Each patient came in for three or four visits, depending on whether they started with No-Interaction (four visits) or Clinical-Interaction (three visits; the initial consent/behavioral and clinical intake sessions were completed during the same visit). Each clinician came in for three visits—depending on interaction order, with their initial behavioral session completed on the same visit as the No-Interaction MRI (since clinicians’ initial behavioral session was shorter in duration than for patients) or immediately before the clinical intake session with the patient (for those starting with Clinical-Interaction). See below for further detail on each session.

#### Initial consent/behavioral session

After informed consent, participants were seated with a pressure cuff wrapped around their left lower leg, level with the gastrocnemius muscle. Participants went through a cuff pain calibration procedure to determine an individual stimulus intensity (pressure) level corresponding to moderate pain (40/100 pain rating). This pressure level was then used for all experimental cuff stimuli for this individual. Patients then had two acupuncture needles inserted on the anterior/distal aspect of the lower thigh, proximal to the cuff, with electrodes attached to each needle. Patients were then familiarized with the anticipation cue and pain stimuli and received six cuff stimuli, three of which were preceded by a visual cue indicating that upcoming evoked pain would be treated with subsensory threshold EA (see below). For these treatment trials, cuff pressure was surreptitiously reduced by 5, 10, and 20% of the target pressure (randomized order) to enhance expectations of treatment benefit, similar to boosting approaches previously used in investigations of the placebo effect (*32*, *50*).

#### Clinical intake

To maximize ecological validity, clinicians were instructed to perform a clinical consultation and intake with the patient “as similarly as possible to your daily practice.” Clinicians were not given restrictions on the duration of the intakes (mean ± SD = 37:40 ± 12:30 min:s, range = 21:32 to 54:40).

#### MRI sessions

Once the patient had been positioned in the MRI scanner (Skyra, 3T, Siemens Medical, Germany), the clinician entered the scanner room and led the patient through the process of acupuncture needling. MRI-compatible titanium needles (0.22 mm in thickness and 40 mm in length; DongBang Acupuncture Inc., Boryeong, Korea) were inserted proximal to the cuff (2 to 3 cm in depth; acupoints ST-34 and SP-10), with MR-compatible electrodes attached to each needle. These acupoints were chosen for their local/segmental effects on a pain source delivered at the calf. Because of hospital policy, the actual needle penetration was performed by a staff acupuncturist with hospital credentials, but under direct supervision of the subject clinician, and evident to the patient. The clinician then attached MRI-compatible electrodes to the needles, and electrodes were connected to an electronic needle stimulation device (2 Hz, 0.1 mA; AS SUPER 4 Digital, Schwa-Medico, Wetzlar, Germany), controlled by the computer running the experimental protocol. The acupuncturist was then positioned in the other MRI scanner (Prisma, 3T, Siemens Medical, Germany), a 1-min walk within the same building. To allow for unimpeded facial coverage for video transfer, both participants were positioned with an adapted coil configuration, using the 64-channel head coil bottom, and a small (4-channel) flex coil wrapped over the participants’ forehead to cover the frontal lobes of the brain. Before the scan, participants were instructed that they would be free to communicate their feelings to the other person nonverbally using facial expressions, as long as they kept their head as still as possible. Before fMRI scanning for the Clinical-Interaction session, the clinician was given the option to “check in” with the patient via the between-scanner audio/video connection to reinforce the clinical relationship.

### Self-report assessments

#### Therapeutic alliance

To assess the therapeutic empathy attributed to clinicians, patients filled out the validated CARE (*24*) scale after the intake and after each MRI visit, while clinicians filled out a modified CARE questionnaire with items phrased from the clinician’s point of view (*51*, *52*). Relational empathy was used as a proxy for therapeutic alliance.

#### Hyperscan relationship scale

To assess ecological validity during MRI hyperscanning, as well as different qualities of the clinical interaction, we created a custom questionnaire to be filled out by patients (9 items, of which 2 were reversed) and clinicians (10 items, of which 2 were reversed) after each MRI visit (VAS, 0 to 10; anchors, “Completely disagree” and “Completely agree”).

The patient scale included the following items: (i) I had frequent eye contact with the acupuncturist. (ii) I felt as if the acupuncturist was in the same room as me. (iii) I felt like I could communicate with the acupuncturist. (iv) I felt comforted by seeing the acupuncturist. (v) I felt discomforted by seeing the acupuncturist. (vi) I felt as if the acupuncturist was really trying to treat my leg pain with electroacupuncture. (vii) The acupuncturist was genuinely concerned for me when I was in pain. (viii) I expressed my feelings to the acupuncturist. (ix) The acupuncturist was emotionally distant.

The clinician scale included the following items: (i) I had frequent eye contact with the patient. (ii) I felt as if the patient was in the same room as me. (iii) I felt like I could communicate with the patient. (iv) I felt comforted by seeing the patient. (v) I felt discomforted by seeing the patient. (vi) I thought my treatment was helping the patients’ pain. (vii) I felt genuine concern for the patient when she was in pain. (viii) I expressed my feelings to the patient. (ix) I felt emotionally distant from the patient. (x) I cared whether I was providing electroacupuncture or not.

#### In-scanner ratings

At the end of each trial, participants used an MRI-compatible button-box to deliver two consecutive ratings (8 s each) on a VAS. Patients rated pain intensity (“how painful was the cuff?” with anchors “no pain” and “most pain imaginable”) and affect related to either receiving treatment (“how did you feel about getting treated with electroacupuncture?” with anchors “extremely negative” and “extremely positive”) or not receiving treatment (“how did you feel about not getting the electroacupuncture?” with anchors “extremely negative” and “extremely positive”). Clinicians rated vicarious pain (“how painful was it for the patient?”) and affect related to either providing treatment (“how did you feel about doing the electroacupuncture?”) or not providing treatment (“how did you feel about not doing the electroacupuncture?”) with anchors “extremely negative” and “extremely positive.”

#### Treatment expectancy

Before the scan at each MRI visit, participants indicated their expectancy of EA treatment efficacy using a 0 to 10 VAS (patient rating: “How much cuff pain relief do you expect to experience while being treated with electroacupuncture?” with anchors “no pain relief” to “complete pain relief”; clinician rating: “How much cuff pain relief do you expect the patient will experience while being treated with electroacupuncture?” with identical anchors).

### Other materials

#### Cameras

For both participants, visual stimuli were projected onto a screen behind the MRI scanner bore, and participants viewed projected video through a mirror. To enable visual communication between the scanners, MRI-compatible cameras (Model 12M, MRC Systems GmbH, Heidelberg, Germany) were attached to the table-mounted mirror with each MRI scanner and manually adjusted to capture the full face. The two-way video stream (20 Hz) was sent over a local network (measured to have consistent <40-ms delay) and recorded for human facial expression artificial intelligence analyses (see below).

#### Microphones

MRI-compatible optical microphones (Fibersound FOM1-MR, Micro Optics Technologies Inc., Cross Plains, WI, USA) were also set up in each MRI scanner to enable verbal communication between scans. To avoid speech-related motion during fMRI, we decided to disallow verbal communication during fMRI scanning.

#### Software for stimulus presentation and signal synchronization

A custom in-house software (C++) was created for synchronizing fMRI scans between MRI scanners, transferring video and audio signals, and tracking the network delay between scanners. One laptop in each MRI scanner controlled the initiation of the fMRI scan acquisition sequence via remote trigger, the video stream, the experimental design visual stimuli, onset/offset of the cuff stimuli via remote trigger, and recording of in-scanner ratings. Both laptops were connected through a local area network. The MRI teams in each control room communicated with one another via phone, and when ready to start, the master computer (patient MRI control room) sent a signal to the slave computer (clinician MRI control room) to initiate the fMRI pulse sequence. Thus, after a lag corresponding to the current network delay (mean ± SD = 81.6 ± 38.1 ms, calculated as a mean of 10 network pings), each computer initiated the fMRI pulse sequence locally. This procedure ensured synchronized timing of the two fMRI time series, video streams, and experimental protocols.

### Statistical analysis

All nonimaging statistical analyses were completed using R (RStudio 1.1.456) and JASP (version 0.10, Jasp Team, Amsterdam, Netherlands). Threshold for statistical significance was set at α = 0.05.

#### Therapeutic alliance

To evaluate whether therapeutic alliance (CARE score) was different between sessions, we performed separate one-way repeated-measures ANOVAs for the patient group and the clinician group, each with three levels (Intake, Clinical-Interaction MRI, and No-Interaction MRI). We then performed follow-up contrasts comparing the different sessions.

#### Influence of therapeutic alliance at the intake on social interaction at the MRI session

To evaluate whether the relationship established during the intake carried over to the Clinical-Interaction MRI, we performed two ANCOVAs (separately for patient-rated and clinician-rated scores), with HRS values at MRI (see the “Hyperscan relationship scale” section above) as the dependent variable, as an indicator of social interaction quality. Therapeutic alliance at intake (CARE_Intake_) was used as a continuous predictor, and HRS Item was used as a categorical predictor to investigate potential differences between items of the HRS scale.

#### Pain and affect

Ratings of cuff pain intensity and affect were analyzed using separate repeated-measures ANOVAs with factors Treatment condition (Treatment and No-Treatment), Clinical context (Clinical-Interaction and No-Interaction), and Order as a between-subjects factor (Clinical-Interaction first and No-Interaction first).

#### Association between social relationship and patient analgesia

To evaluate whether differences in the social interaction between dyads were associated with analgesia, we performed an ANCOVA with analgesia (Pain_Treat-NoTreat_) as the dependent variable, HRS values (Patient-rated) as a continuous predictor and Clinical context (Clinical-Interaction and No-Interaction) and HRS Item (see the “Hyperscan relationship scale” section above) as categorical predictors.

#### Treatment expectancy

To evaluate whether prior expectancy of therapeutic efficacy predicted treatment-related pain relief, we calculated Pearson’s correlation coefficients between expectancy as rated by the patient and the clinician before scanning versus analgesia (mean Pain_Treat-NoTreat_) during scanning.

#### Facial expression analyses

Facial expressions during fMRI scanning were analyzed using automated facial feature extraction (Affectiva, Cambridge, MA). The Affectiva Facial Expression Analysis algorithm is based on the Emotional Facial Action Coding System (*53*) and trained on ~8 million images and videos of faces. Because of limited field of view in forehead and chin regions for some participants, we were able to fully analyze patient data from 24 dyads and clinician data from 21 dyads (17 dyads had adequate data for both patient and clinician data). The intact data represent a relatively equal group distribution of participants (*n* = 8 from Social Interaction and *n* = 9 from No-Interaction) and include participants comparable in patient age (mean ± SD: limited sample, 37.5 ± 10.6; full sample, 39.95 ± 10.93), clinician age (limited sample, 42.75 ± 12.42; full sample, 44.32 ± 12.81), and patients’ clinical pain levels (limited sample, 46.99 ± 18.93; full sample, 44.10 ± 19.82), which supports the contention that this subsample is likely to be representative of the total sample from which other outcomes are reported. For the Affectiva algorithm, 33 facial landmarks are initially identified, which were used to estimate 21 facial action units. These units were then mapped onto seven basic emotional expressions (joy, fear, disgust, sadness, anger, surprise, and contempt) and two core expressions (valence and engagement). We calculated these nine expressions frame by frame and averaged across each trial duration (separately for anticipation and pain/treatment phases).

##### Overall mirroring

Behavioral mimicry such as the mirroring of facial expressions is thought to be fundamental for social development (*54*, *55*) and a cornerstone of the establishment and maintenance of human bonds (*56*, *57*), including in the patient-clinician interaction (*58*). As the specific facial expressions mirrored can be variable across individuals and interactions, we decided to investigate correspondence within each dyad and across different expressions. We first calculated the difference in each expression between anticipation of Pain/Treatment relative to Pain/No-Treatment. Using these difference scores, we then calculated a Pearson’s correlation coefficient between the patient and the clinician of each dyad. This coefficient was then Fisher’s *r*-to-*z* transformed and used as a metric of each dyad’s overall facial mirroring. We then investigated whether facial mirroring was associated with therapeutic alliance (CARE scores) and analgesia.

#### fMRI analysis

##### Treatment-related differences in pain-related brain activation

Details on MRI acquisition and preprocessing are described in Supplementary Materials and Methods. For all whole-brain group fMRI analyses, significance testing was performed using FSL FLAME 1+2 with cluster correction for multiple comparisons (*z* = 2.3, α = 0.05) (*59*). To investigate treatment-related differences in brain response during pain, we first performed single-subject first-level GLM analyses using FILM with local autocorrelation correction (*60*). For each of the two runs (six trials each), we modeled periods corresponding to pain stimulation (Treat and NoTreat) as regressors. In the same design matrix, we also modeled ratings periods and the six motion parameter time series as regressors of no interest. We computed two bidirectional contrasts: Pain_Treat_-Rest, Pain_NoTreat_-Rest. In second-level fixed-effects analyses, we averaged these contrast parameter estimates across both runs and both visits (Clinical-Interaction and No-Interaction) for each patient. The resulting contrast parameter estimate maps were then passed up to a group analysis where a whole-brain group mean was calculated for all patients.

##### Regression with analgesia

To investigate brain regions where treatment-related change in BOLD contrast correlated with analgesia, we performed a whole-brain regression GLM using each patient’s mean analgesia (Pain_Treat_ – Pain_NoTreat_) ratings as a regressor of interest.

##### Regression with patient-clinician correspondence in pain/vicarious pain

To investigate brain regions where treatment-related change in the clinicians’ fMRI response during the Pain/Treatment period correlated with clinicians’ accuracy in evaluating the patients’ pain, we performed a whole-brain regression GLM. This analysis used clinicians’ brain treatment-related activation (i.e., Treat-NoTreat) and the patient-clinician trial-by-trial correspondence in pain/vicarious pain ratings for each dyad (see above for a detailed description of this metric) as a regressor of interest.

##### Overall brain response to anticipation and pain

Patient-clinician concordance related to therapeutic alliance and pain outcomes could be driven by social interaction during the anticipation of both treated and nontreated pain. Therefore, we first calculated overall brain response to anticipation of pain irrespective of Treat/NoTreat conditions, followed by a group conjunction between patients and clinicians, to identify ROIs for concordance analyses.

Specifically, single-subject GLM analyses were performed using FILM with local autocorrelation correction. Similar to above, for each of the two runs, we modeled periods corresponding to anticipation of pain and pain stimulation as regressors. We also modeled ratings periods and the six motion parameter time series as regressors of no interest. We computed bidirectional contrasts for Anticipation-Rest and Pain-Rest. The Rest control period comprised the periods outside anticipation, pain stimulation, and ratings. In second-level fixed-effects analyses, we averaged these contrast parameter estimates across both runs and both visits (Clinical-Interaction and No-Interaction) for each individual. We then passed the resulting contrast parameter estimate maps up to group analyses (separately for patients and clinicians), indicating overall response to (i) anticipation of pain (patients) and preparing to treat/not treat (clinicians) and (ii) pain (patients) and observing pain and treating/not treating (clinicians). To identify shared activation between patients and clinicians during the anticipation phase, for ROI identification for concordance analyses, we first performed a conjunction of the minimum statistic between these two maps. This group conjunction map was corrected for multiple comparisons using false discovery rate (α = 0.05). We then intersected this whole-brain map with a priori structural ROIs involved in social mirroring, empathy, and theory of mind (bilateral “Insular Cortex”, “Inferior Frontal Gyrus, pars triangularis”, and “Inferior Frontal Gyrus, pars Opercularis”, from the Harvard-Oxford Cortical atlas, p>5%; and bilateral “TPJa” and “TPJp” from the Mars TPJ connectivity-based parcellation atlas, p>5%) to yield more specific functional ROIs for use in concordance analyses.

##### Patient-clinician dynamic concordance in brain activity

Given the structured experimental design with designated periods with visual cues for anticipation, pain stimulation/treatment and ratings, it was pertinent to properly model this structure in a multilevel approach to minimizing “pseudo-concordance” driven more by the shared experimental structure across individuals than social interaction itself (*25*). We therefore decided to test for patient-clinician dynamic concordance across trials after first-level individual modeling, instead of a model-free volume-by-volume level correlation across the entire session, which may have been more susceptible to externally derived pseudo-concordance influenced by shared experimental structure, as well as potential confounds introduced by individual differences in hemodynamic response function (*61*, *62*). We also note that, while the assessment of trial-by-trial dynamics, using block temporal units spanning several seconds may be less appropriate for signals with high-frequency temporal dynamics such as electrical potentials measured by scalp electroencephalography, it is commonly considered appropriate for the slower-frequency BOLD signal dynamics assessed by fMRI (*63*, *64*).

To assess dynamic concordance in brain activity between patients and clinicians, we first performed two first-level GLMs (one for each fMRI scan run), with each trial (anticipation period) as a separate regressor (fig. S7). We also modeled each pain period as a separate regressor of no interest. This produced a total of 12 pain anticipation parameter estimate maps (across both runs) for each individual. We then extracted the mean Zstat value from each individual’s rTPJ for each of the 12 anticipation trials, as defined by the group conjunction map intersected with the anatomical ROI. This same ROI was used for all dyads, after individual data were registered to MNI152 standard space. For each dyad, we performed a second-level whole-brain regression analysis of the clinician’s brain, using the trial-by-trial rTPJ Zstat values from the patient as a regressor and vice versa. Thus, we obtained a whole-brain map for each individual showing regions dynamically concordant (across trials) with the dynamics of the partner’s rTPJ response throughout the interaction. Next, we performed a whole-brain group contrast between Clinical-Interaction and No-Interaction to investigate regions where dynamic concordance was increased by Clinical-Interaction.

##### Mediation analysis

Last, we explored whether treatment-induced change in patients’ brain response during pain reflecting analgesia (i.e., Treat-NoTreat) mediated the influence of brain concordance on analgesia ratings. We decided to focus on rTPJ-to-rTPJ concordance, as this metric was correlated with analgesia. We first extracted the mean Zstat value from the rTPJ region of each clinician’s whole-brain concordance map with the patient’s rTPJ, as a metric of each dyad’s rTPJ-rTPJ concordance, which was then used as the independent variable. For the mediator variable, we focused on the vlPFC, as this region is a key region for social mirroring (*12*, *65*–*68*) and has been implicated in psychosocial and placebo analgesia (*30*, *69*–*72*). The vlPFC ROI was chosen on the basis of an intersection between the Pain_Treat-NoTreat_ regression map and an anatomical mask (Inferior Frontal Gyrus, pars triangularis and pars opercularis, combined mask, *P* > 30%). We extracted the mean Zstat value from the vlPFC_Treat-NoTreat_ Zstat ROI from each patient, which we then used as a mediator variable in further analyses. In addition, since the Pain_Treat-NoTreat_ contrast was also increased for patients in brain regions beyond vlPFC (e.g., TPJ, dlPFC, STS, and mPFC), we also explored whether these regions mediated the association between concordance and analgesia. Each patient’s (Pain_Treat-NoTreat_) rating difference was used as the dependent variable. We used the R package “Mediation” for mediation analyses (*73*). We tested for statistical significance using a boot strapping approach (1000 iterations, α = 0.05) and considered the mediation significant if the total indirect effect (*a* * *b*) was statistically significant, while the previously significant direct effect (path *c*) became nonsignificant after controlling for the mediator (*c*′) (*74*).

## Supplementary Material

abc1304_SM.pdf

## SUPPLEMENTARY MATERIALS

Supplementary material for this article is available at http://advances.sciencemag.org/cgi/content/full/6/43/eabc1304/DC1

## Appendix

View/request a protocol for this paper from *Bio-protocol*.
