# Supplementary material for: Dynamic brain-to-brain concordance and behavioral mirroring as a mechanism of the patient-clinician interaction
Source: Sci Adv. 2020 Oct 21;6(43):eabc1304. doi: 10.1126/sciadv.abc1304 (PMC7577722; doi:10.1126/sciadv.abc1304)
Supplement: abc1304_SM.pdf [file abc1304_SM.pdf]

Science Advances
AAAS

advances.sciencemag.org/cgi/content/full/6/43/eabc1304/DC1

Supplementary Materials for

Dynamic brain-to-brain concordance and behavioral mirroring as a mechanism of the patient-clinician interaction

Dan-Mikael Ellingsen*, Kylie Isenburg, Changjin Jung, Jeungchan Lee, Jessica Gerber, Ishtiaq Mawla, Roberta Sclocco, Karin B. Jensen, Robert R. Edwards, John M. Kelley, Irving Kirsch, Ted J. Kaptchuk, Vitaly Napadow

*Corresponding author. Email: d.m.ellingsen@psykologi.uio.no

Published 21 October 2020, *Sci. Adv.* **6**, eabc1304 (2020)
DOI: 10.1126/sciadv.abc1304

This PDF file includes:

Supplementary Methods and Materials
Figs. S1 to S8
References

Supplementary Methods and Materials

Stimuli

Evoked cuff pain

Deep-tissue pain was applied using the Hokanson Rapid Cuff Inflator (D. E. Hokanson, Inc., Bellevue, WA, USA). Compared to cutaneous quantitative sensory testing (QST) techniques (e.g., contact heat), deep sustained pain better mimics clinical pain (*75*, *76*), thus providing a more clinically-relevant measure. Unlike more superficial methods of evaluating mechanical sensitivity, cuff pain responses are only marginally affected by sensitization or desensitization of the skin, indicating that this procedure primarily assesses sensitivity in muscle and other deep tissues (*77*–*80*). Such cuff pressure algometry is a recently characterized method that is now included in many QST evaluations (*77*, *78*, *81*). We have considerable experience applying these techniques in chronic pain patients, with and without neuroimaging, and have found that even subjects with severe fibromyalgia are generally able to tolerate these procedures without any lasting discomfort (*82*, *83*). The cuff was attached to the patient's left lower leg prior to scanning and was inflated for 15 s duration trials during the experiment.

Electroacupuncture

Our decision to use electroacupuncture applied by acupuncturists for evoked cuff pain as an experimental model for dyadic interaction with pain therapy was motivated by our aim to investigate both a consultation/intake and actual treatment with measurable pain outcomes. As opposed to many other pain therapies, this electroacupuncture/cuff pain model enabled us to incorporate acute pain therapy in the MRI environment, with acupuncturists remotely applying treatment relevant to their clinical practice, thus increasing ecological validity while maintaining experimental control.

Prior to experimental testing, patients had acupuncture needles (0.20 mm diameter, 25 cm length, Asiamed gauge) inserted with approximately 2-3cm depth at acupoints ST-34 and SP-10 on the anterior/distal aspect of the lower thigh, proximal to the cuff, with electrodes attached to each needle. As we were interested in the influence of psychosocial aspects such as therapeutic alliance, and not specific acupuncture effects, on pain outcomes, we wanted to avoid the need for authorized deception in clinicians' IRB consent form, while maintaining clinicians' belief they were indeed providing potentially effective treatment during a verum condition. We therefore included trials with both verum and sham electro-acupuncture in a double-blind manner. We used a minimal sub-sensory threshold level (0.1 mA) for verum trials in order to avoid unblinding patients due to any sensory feedback from the electrical stimulation. For this intensity level and duration, the verum condition was very unlikely to have had any significant physiological effect. Thus, verum/sham electro-acupuncture was administered in a double-blind manner, in a pseudorandomized order across trials. Clinicians were instructed to press-and-hold one button for applying treatment, and another ("inactive") button for No-treatment.

We did not hypothesize differences in pain for verum vs sham electro-acupuncture treatment, and indeed, a paired t-test comparing patient-rated pain intensity between verum and sham trials did not suggest a difference in pain intensity (t=0.83, P=0.42). Furthermore, an equivalence test (R package TOSTER, ver. 0.3.4, TOSTpaired function, $\alpha = 0.05$, equivalence bounds: d_z= -0.5 – 0.5 (*84*)) confirmed that the difference between verum and sham was statistically equivalent to zero (t=2.75, P=0.005). Finally, there was not a differential association between baseline expectation and analgesia, when comparing Sham-NoTreat (r=-0.14, P=0.41) and Verum-NoTreat (r=-0.09, P=0.60). Consequently, we pooled verum/sham trials as 'Treatment' in further statistical analyses, and interpreted intra-individual differences between 'Treatment' and 'No-Treatment' trials as psychosocially induced pain relief ('analgesia').

MRI acquisition and preprocessing

MRI acquisition

Blood oxygen level-dependent (BOLD) fMRI data were collected from each scanner (Patient scanner: Siemens 3T Skyra; Clinician scanner: Siemens 3T Prisma) using a whole brain, simultaneous multi-slice, T2*-weighted gradient echo BOLD echo-planar imaging pulse sequence (repetition time = 1250 ms, echo time = 33 ms, flip angle = 65°, voxel size = 2 cm isotropic, number of slices = 75, Simultaneous Multi-Slice factor = 5). A high-resolution structural volume (multi-echo MPRAGE) was collected to facilitate anatomical localization and spatial registration of individual fMRI-BOLD volumes to MNI152 standard space (repetition time = 2530 ms, echo time = 1.69 ms, flip angle = 7°, voxel size = 1 mm isotropic). Importantly, to enable a full-face view of each participant for better facial expression tracking by research subjects and the facial expression digitization software (see below), we combined the occipital/bottom portion of a 64 Channel head coil with a flex coil (4 channel) attached to the forehead.

fMRI preprocessing

Preprocessing of individual fMRI datasets was carried out using tools from FMRIB's Software Library (FSL, v6.0.0; www.fmrib.ox.ac.uk/fsl), and included the following steps: slice-timing correction, motion correction (MCFLIRT) (*85*), correction of spatial inhomogeneity (TOPUP) (*86*, *87*), nonbrain tissue removal (BET) (*88*), spatial smoothing (full width at half maximum = 4mm), temporal high-pass filtering (f=0.011 Hz as computed by FSL's cutoffcalc), and grand-mean intensity normalization by a single multiplicative factor. For each subject, both runs were realigned (6 degrees of freedom) to a common space (7th volume of the first run) before the first-level general linear model (GLM) analyses. The transformation matrix for registration

between functional and high-resolution anatomical volumes was calculated using Boundary Based Registration (bbregister, Freesurfer, v6.0.0 (*89*)). Two participants had one of their two fMRI runs excluded from analysis due to excessive head motion, based on the following exclusion criteria: 1) >2° head rotation in any direction, and 2) >2 mm frame-by-frame displacement. After excluding these data, mean head rotation was 0.05±0.02 (mean±SD) and mean frame-by-frame displacement was 0.13±0.05. An unpaired t-test indicated higher frame-by-frame displacement for patients (0.15±0.05) relative to clinicians (0.11±0.04, t=4.32, P<0.001), but there was no significant group difference for rotation (t=1.74, P=0.09). For registration from structural to standard space (Montreal Neurological Institute, MNI, 152), we used FSL's Linear registration tool (FLIRT, 12 degrees of freedom) (*85*, *90*), followed by FSL's non-linear registration tool (FNIRT) (*91*). All single-subject analyses were performed in functional space, and then registered to MNI152 standard space before dyadic and group analyses.

Supplementary Figures

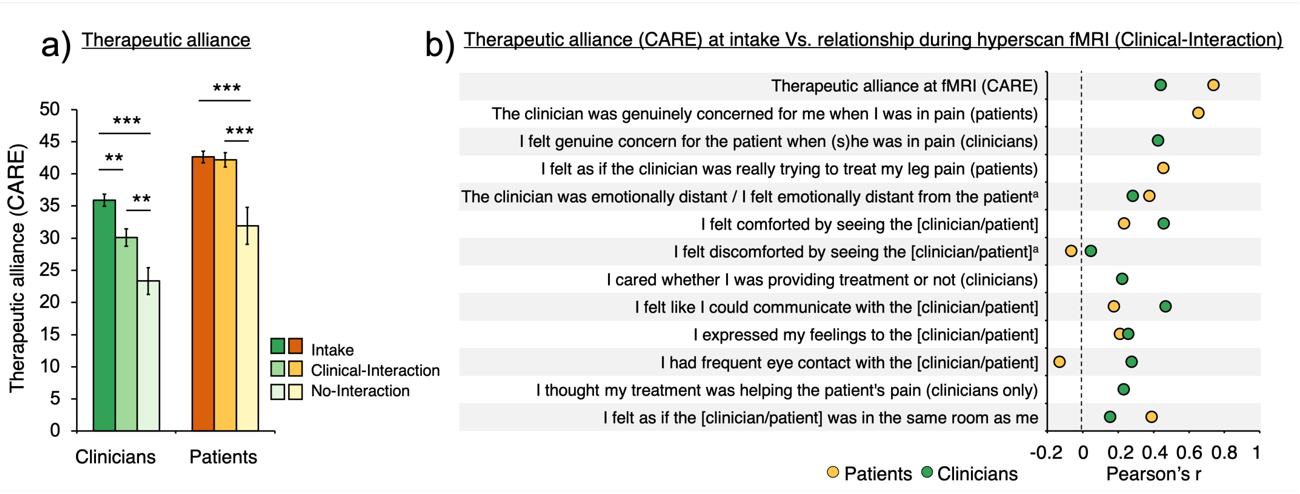

Fig. S1 | Self-reported patient-clinician therapeutic alliance (CARE) across sessions. a) Similarly to patients (see main Article), clinicians reported different levels of therapeutic alliance (CARE scores)

depending of the context of the dyadic clinical interaction ($F(1.82,25.42)=12.83$, $P<0.001$, $\eta_p^2=0.48$). Specifically, the No-interaction MRI context (mean±SD=23.56±7.94) was rated lower than both Intake (35.69±4.08, t=5.95, $P<0.001$, Cohen's *d*=1.49, 95% Confidence Interval [CI]=6.63,17.62) and Clinical-Interaction MRI (30.25±5.89, t=2.84, P=0.01, *d*=0.71, CI=0.34,13.03) contexts. Furthermore, therapeutic alliance at Intake was rated higher than at Clinical-Interaction MRI (t=3.11, P=0.01, *d*=0.78, CI=0.72,10.16). There was no significant effect of order for neither patient-rated CARE ($F(1.34,18,76)=1.07$, P=0.34) $\eta_p^2=0.07$) nor clinician-rated CARE (F(1.82,25.42), P=0.63, $\eta_p^2=0.03$). b) An ANCOVA indicated that higher therapeutic alliance (CARE scores) at intake positively predicted evaluations of the relationship ('HRS score') at the subsequent Clinical-Interaction MRI, across a range of items related to the relationship and social interaction. This association was evident for both for the patient-rated ($F(1,144)=9.33$, P=0.003 $\eta_p^2=0.06$) and the clinician-rated ($F(1,160)=12.24$, $P<0.001$, $\eta_p^2=0.07$) scales, indicating the relationship and rapport established at the intake was successfully carried over to the Clinical-Interaction MRI, which was completed on a separate day. There were no main effects or statistical interactions involving 'HRS Item' for neither patients nor clinicians (P's>0.39), suggesting the association between therapeutic alliance at intake Vs relationship at MRI, as well as HRS scores overall, was not different depending on HRS items. Error bars represent Standard error of the mean; [a]Reversed score; CARE=Consultation And Relational Empathy scale.

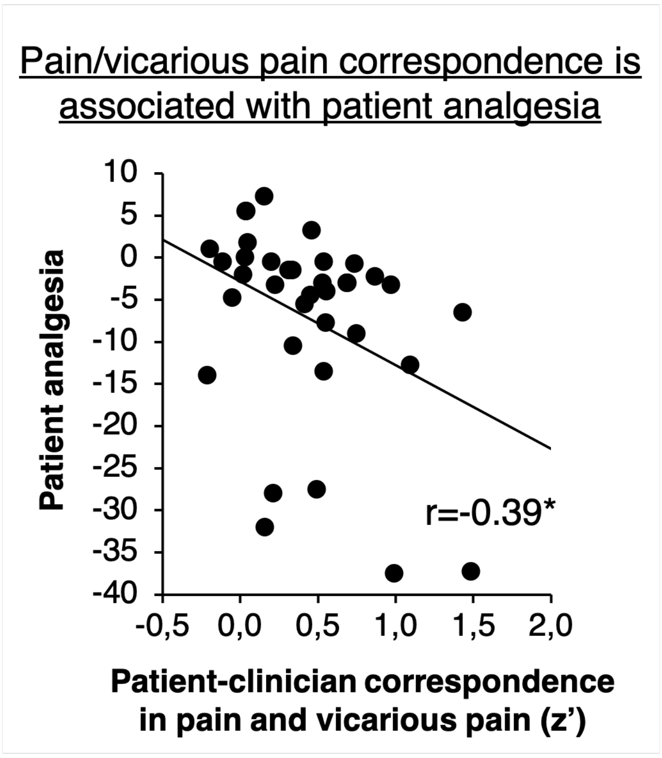

Fig. S2: A correlation between patient analgesia (ΔPain, Treat-NoTreat) and patient-clinician correspondence in pain / vicarious pain ratings indicated that, for dyads where the clinician was better able to accurately estimate their patient's pain, the patient reported stronger treatment analgesia (r=-0.39, P=0.017). Patient-clinician correspondence in pain and vicarious pain was estimated by a Pearson's correlation coefficient across trials for the patient's rated pain and the clinician's vicarious pain rating for each dyad, which was then r-to-z transformed.

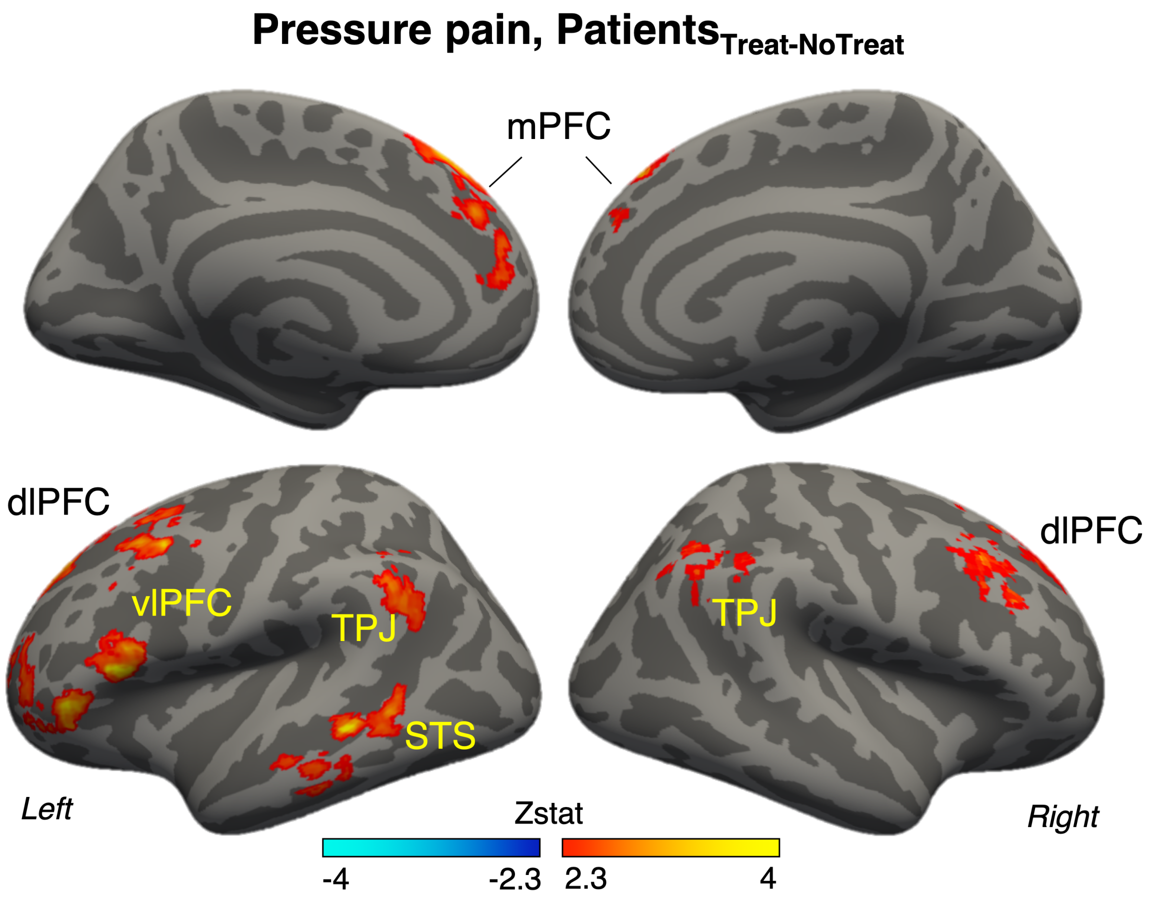

Fig. S3 | Group map displaying areas where patients showed significant treatment-related (Treat – NoTreat) increase in brain response to pressure pain, thresholded at Z=2.3, P<0.05, cluster-corrected for multiple comparisons. A whole-brain GLM demonstrated increased fMRI activation of bilateral vlPFC, TPJ, dlPFC, and mPFC, in addition to left STS for treated, relative to nontreated, pain. There were no significant effects in the opposite direction (NoTreat – Treat). This is consistent with a recent meta-analysis of experimental fMRI studies investigating placebo analgesia (*92*), showing that while placebo analgesia was seen in all studies, there was only a marginal BOLD reduction of pain-processing circuitry (*39*). mPFC=medial Prefrontal Cortex; dlPFC=dorsolateral Prefrontal Cortex; vlPFC=ventrolateral Prefrontal Cortex; TPJ=Temporoparietal Junction; STS=Superior Parietal Junction.

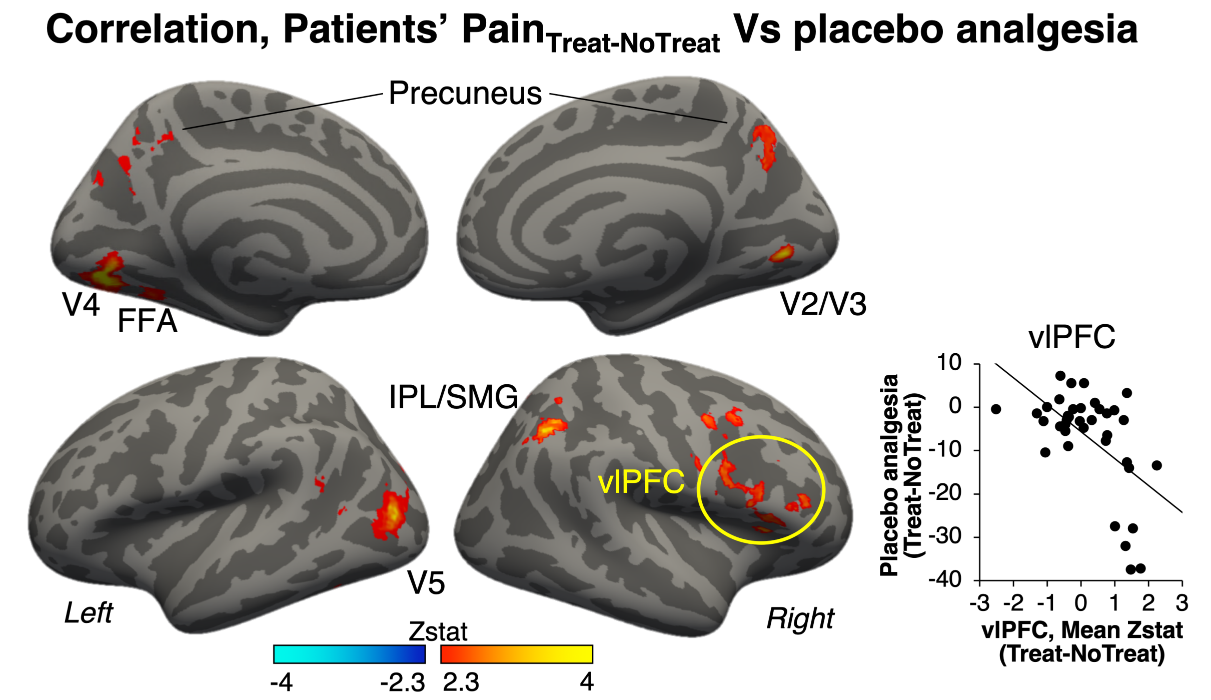

Fig. S4 | Group map showing areas where stronger treatment-related increases (Treat-NoTreat) in patients' brain response to pressure pain was significantly associated with stronger analgesia (NoTreat-Treat, pain ratings), as indicated by a whole-brain regression analysis. The displayed group map was thresholded at Z=2.3, P<0.05, cluster-corrected for multiple comparisons. V2-5=Visual areas 1-5; FFA=Fusiform Face Area; IPL=Inferior Parietal Lobule; SMG=Supramarginal Gyrus; vlPFC=ventrolateral Prefrontal Cortex.

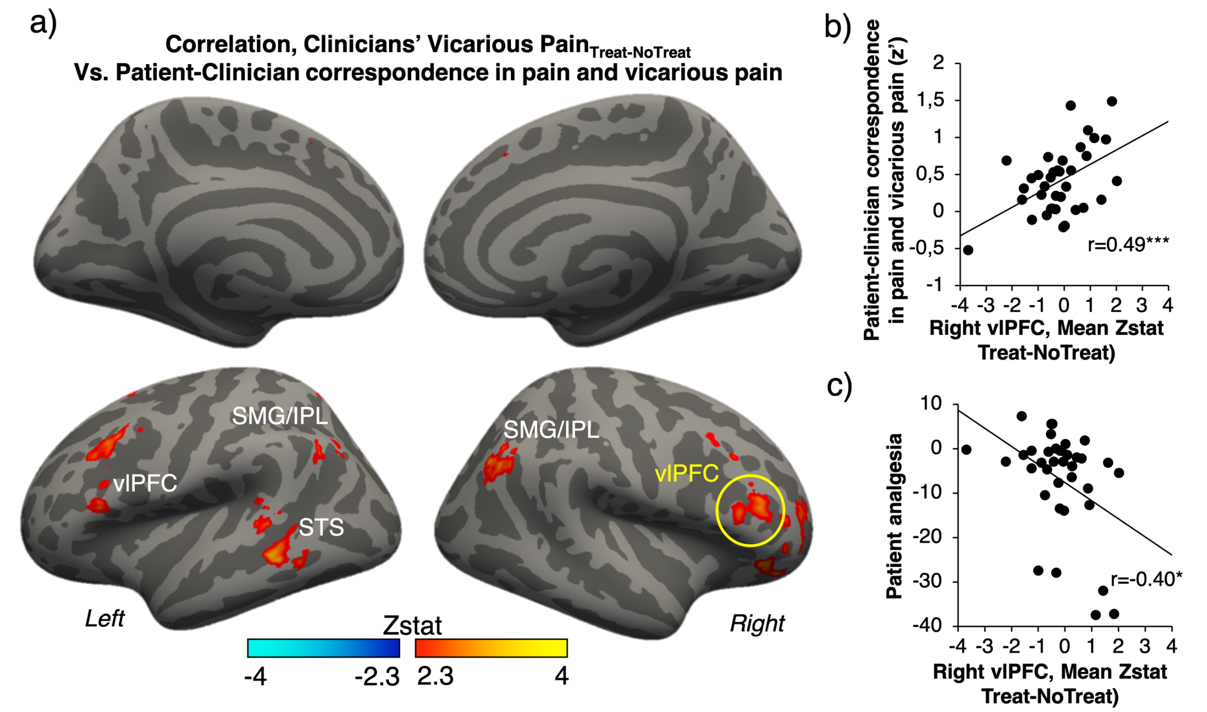

Fig. S5: Investigation of clinicians' brain responses underpinning pain / vicarious pain correspondence. a) A whole-brain regression analysis indicated that clinicians who were better able to accurately estimate their patient's pain intensity (i.e. patient-clinician correspondence in pain and vicarious pain ratings) demonstrated stronger activation of social mirroring circuitry including vlPFC, SMG/IPL, and STS, during provision of Treatment relative to No Treatment (Treat-NoTreat). The displayed group map was thresholded at Z=2.3, P<0.05, cluster-corrected for multiple comparisons. b) Mean zstat values (Vicarious pain (Treat-NoTreat)) from the right vlPFC were extracted to illustrate this relationship. c) Treatment-related activation in the clinician's vlPFC was also correlated with patient analgesia. Specifically, for dyads in which the clinician showed a more positive fMRI response for right vlPFC during treatment relative to no treatment, their dyad-matched patient reported stronger treatment analgesia. vlPFC=ventrolateral Prefrontal Cortex; SMG=Supramarginal Gyrus; IPL=Inferior parietal lobule; STS=Superior Temporal Sulcus; *p<0.05; ***p<0.005.

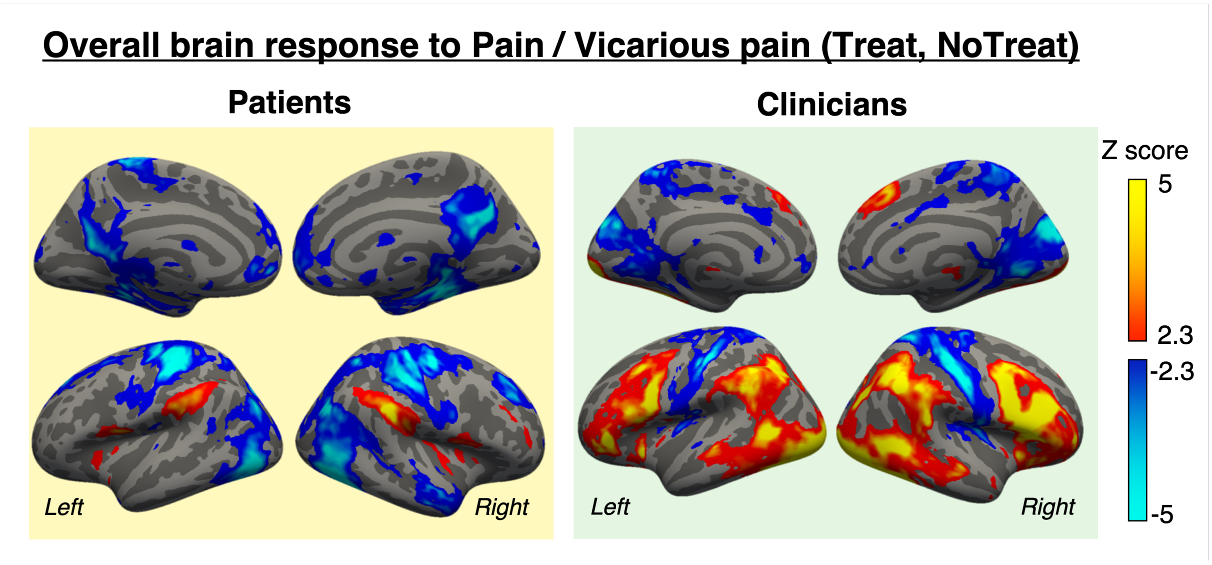

Fig. S6 | Group maps showing overall response to pain (patients, left) and vicarious pain (clinicians, right), collapsed over Treat/NoTreat conditions. The displayed group maps were thresholded at Z=2.3, P<0.05, cluster-corrected for multiple comparisons.

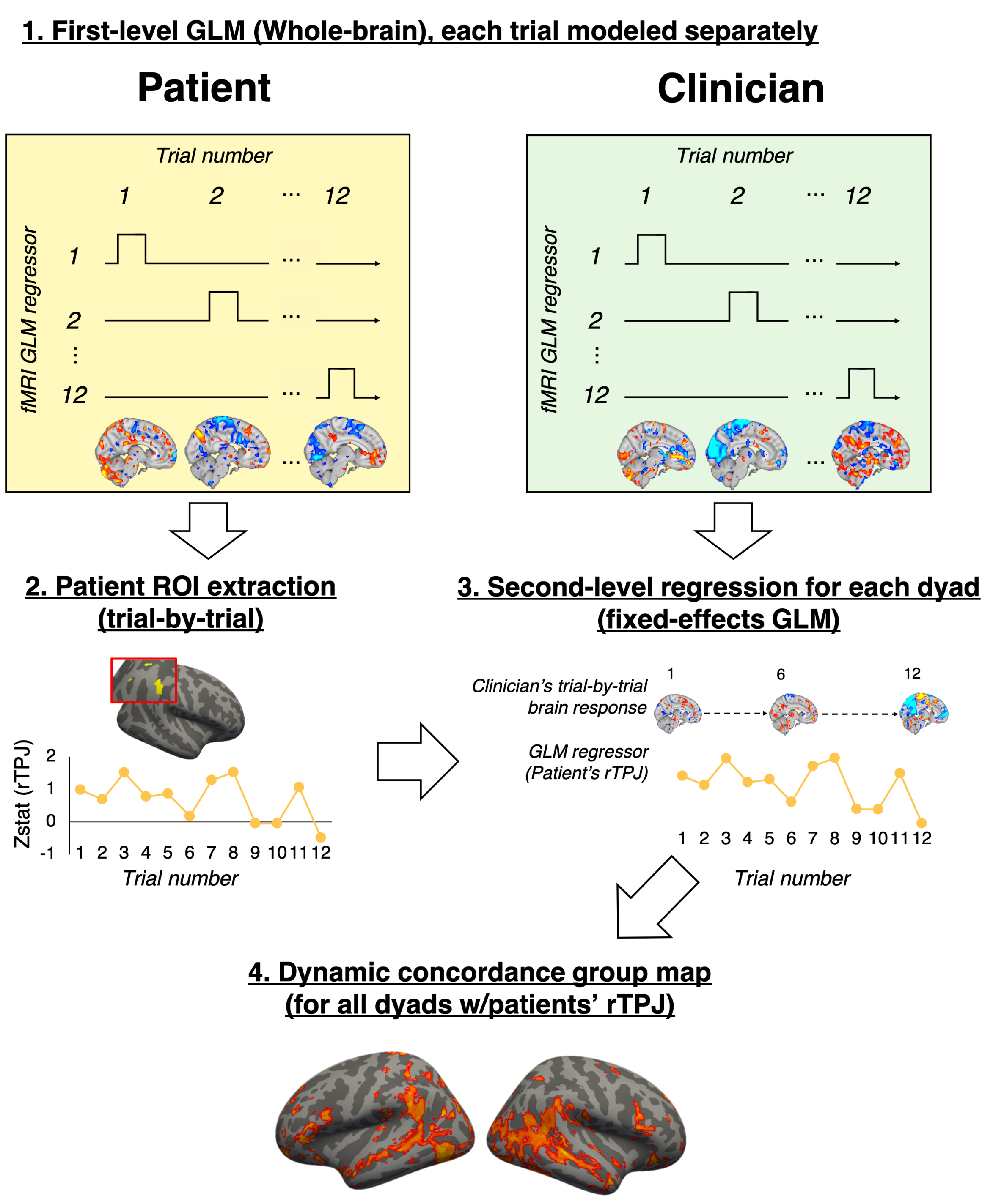

Fig. S7 | Dynamic concordance analysis pipeline. 1. To investigate dynamic (time-varying) concordance between patients and clinicians, we first performed a first-level GLM for each subject, where each trial (anticipation phase) was modeled as a separate regressor. This resulted in 12 voxel-wise whole-brain maps (one per trial). 2. For each trial, mean Zstat values were extracted from the patient's social mirroring ROIs (e.g. rTPJ), as identified by a patient-clinician conjunction analysis (see Fig. a). 3. These ROI scores were then used as a regressor in a second-level fixed-effects GLM for the

clinician's trial-by-trial brain response during anticipation, which produced a whole-brain map of regions where the clinician's brain response showed dynamic (time-varying) concordance with the patient's social mirroring circuitry (e.g. rTPJ). 4. These steps were taken for each individual dyad, and dynamic concordance maps across dyads were then combined for group analyses. ROI=Region of Interest; rTPJ= right Temporoparietal Junction.

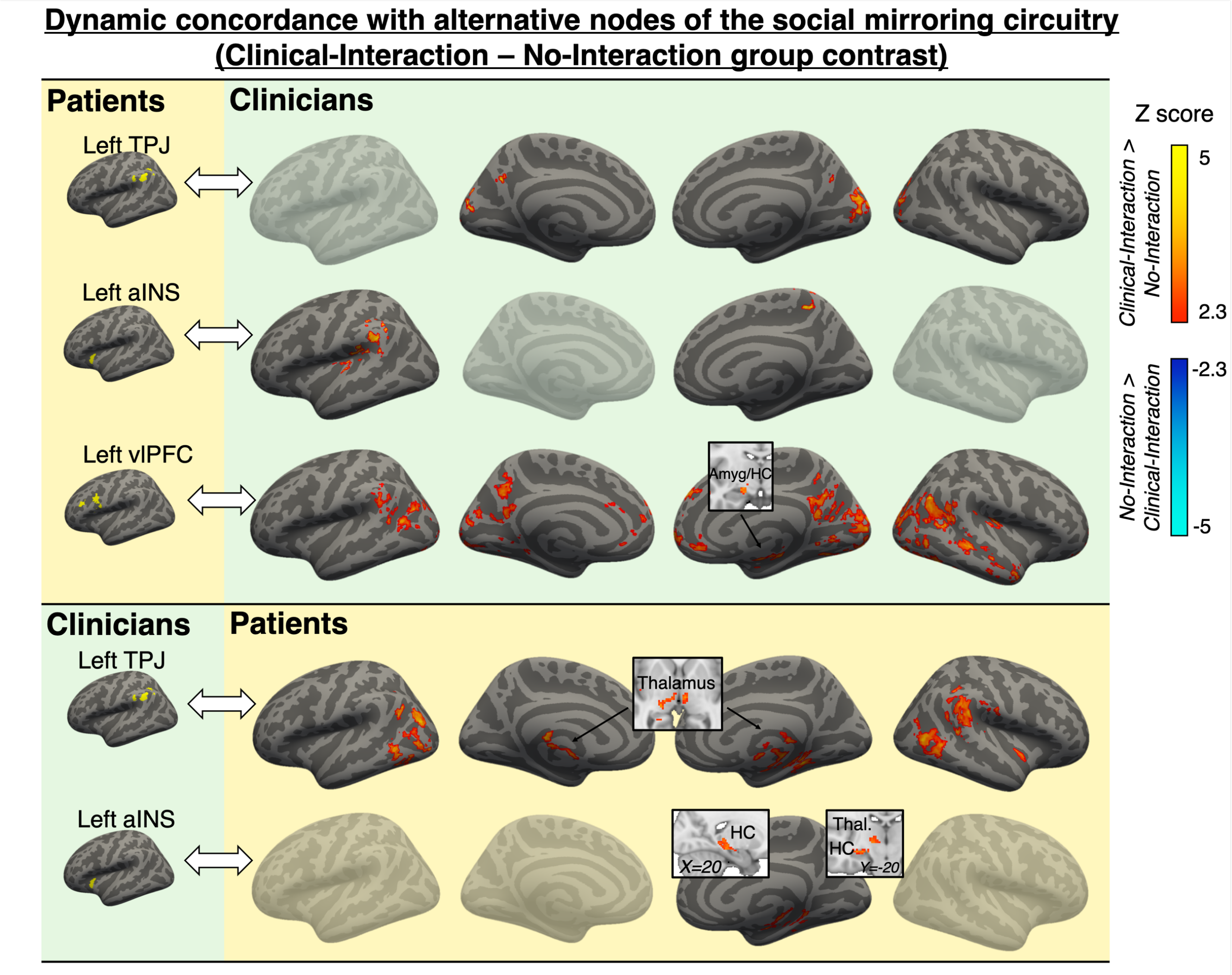

Fig. S8 | Dynamic concordance with alternative nodes of the social mirroring circuitry. Each row shows a group contrast (Clinical-Interaction – No-Interaction) of dynamic concordance with different social mirroring ROIs from the partner, as identified by the patient-clinician conjunction group analysis. The top 3 rows show clinician whole-brain concordance with patients' social mirroring ROIs. Patients' left TPJ showed increased concordance with clinicians' precuneus and medial visual cortex for Clinical-Interaction relative to No-Interaction. For the same contrast, patients' left aINS showed increased concordance with clinicians' left TPJ, left posterior insula, and right S1. Left vlPFC showed increased concordance with clinicians' left TPJ, right amygdala/hippocampus, bilateral STS, right mid/posterior insula, vmPFC/pregenual ACC (pgACC), dmPFC, lateral/medial visual cortex, and precuneus. The bottom 2 rows show patient whole-brain concordance with clinicians' social mirroring ROIs. Clinicians' left TPJ showed increased concordance with patients' right TPJ, right aINS, right STS, lateral visual

cortex, and bilateral thalamus for Clinical-Interaction relative to No-Interaction. Left aINS showed increased concordance with the right HC and the right thalamus. None of these contrasts showed significant differences in the opposite direction (No-Interaction – Clinical-Interaction). No other nodes of the social mirroring circuitry showed differences in concordance between Clinical-Interaction and No-Interaction. The displayed group maps were thresholded at Z=2.3, P<0.05, cluster-corrected for multiple comparisons. There were no significant effects in the opposite direction (No-Interaction – Clinical-Interaction). TPJ=Temporoparietal Junction; aINS=anterior Insula; vlPFC=ventrolateral Prefrontal Cortex; Amyg=Amygdala; HC=Hippocampus.

REFERENCES AND NOTES

1. R. Grob, G. Darien, D. Meyers, Why physicians should trust in patients. *JAMA* **321**, 1347–1348 (2019).

2. K. Whetten, J. Leserman, R. Whetten, J. Ostermann, N. Thielman, M. Swartz, D. Stangl, Exploring lack of trust in care providers and the government as a barrier to health service use. *Am. J. Public Health* **96**, 716–721 (2006).

3. T. J. Kaptchuk, J. M. Kelley, L. A. Conboy, R. B. Davis, C. E. Kerr, E. E. Jacobson, I. Kirsch, R. N. Schyner, B. H. Nam, L. T. Nguyen, M. Park, A. L. Rivers, C. McManus, E. Kokkotou, D. A. Drossman, P. Goldman, A. J. Lembo, Components of placebo effect: Randomised controlled trial in patients with irritable bowel syndrome. *BMJ* **336**, 999–1003 (2008).

4. P. H. Ferreira, M. L. Ferreira, C. G. Maher, K. M. Refshauge, J. Latimer, R. D. Adams, The therapeutic alliance between clinicians and patients predicts outcome in chronic low back pain. *Phys. Ther.* **93**, 470–478 (2013).

5. M. E. Suarez-Almazor, C. Looney, Y. Liu, V. Cox, K. Pietz, D. M. Marcus, R. L. Street Jr, A randomized controlled trial of acupuncture for osteoarthritis of the knee: Effects of patient-provider communication. *Arthritis Care Res.* **62**, 1229–1236 (2010).

6. L. Dyche, D. Swiderski, The effect of physician solicitation approaches on ability to identify patient concerns. *J. Gen. Intern. Med.* **20**, 267–270 (2005).

7. A. H. Kamal, J. H. Bull, S. P. Wolf, K. M. Swetz, T. D. Shanafelt, K. Ast, D. Kavalieratos, C. T. Sinclair, A. P. Abernethy, Prevalence and predictors of burnout among hospice and palliative care clinicians in the U.S. *J. Pain Symptom Manage.* **51**, 690–696 (2016).

8. D. Musa, R. Schulz, R. Harris, M. Silverman, S. B. Thomas, Trust in the health care system and the use of preventive health services by older black and white adults. *Am. J. Public Health* **99**, 1293–1299 (2009).

9. H. Leis, P. Garg, I. Soh, “Right place, right time: Marketplace responses to the health information needs of vulnerable consumers: Final report” (Oliver Wyman, 2017); https://owy.mn/3cdsuRo.

10. T. J. Kaptchuk, F. G. Miller, Placebo effects in medicine. *N. Engl. J. Med.* **373**, 8–9 (2015).

11. D. Bzdok, L. Schilbach, K. Vogeley, K. Schneider, A. R. Laird, R. Langner, S. B. Eickhoff, Parsing the neural correlates of moral cognition: ALE meta-analysis on morality, theory of mind, and empathy. *Brain Struct. Funct.* **217**, 783–796 (2012).

12. K. B. Jensen, P. Petrovic, C. E. Kerr, I. Kirsch, J. Raicek, A. Cheetham, R. Spaeth, A. Cook, R. L. Gollub, J. Kong, T. J. Kaptchuk, Sharing pain and relief: Neural correlates of physicians during treatment of patients. *Mol. Psychiatry* **19**, 392–398 (2014).

13. E. Redcay, L. Schilbach, Using second-person neuroscience to elucidate the mechanisms of social interaction. *Nat. Rev. Neurosci.* **20**, 495–505 (2019).

14. J. Levy, A. Goldstein, R. Feldman, The neural development of empathy is sensitive to caregiving and early trauma. *Nat. Commun.* **10**, 1905 (2019).

15. U. Hasson, C. D. Frith, Mirroring and beyond: Coupled dynamics as a generalized framework for modelling social interactions. *Phil. Trans. R. Soc.Lond. B Biol. Sci.* **371**, 20150366 (2016).

16. Z. E. Imel, J. S. Barco, H. J. Brown, B. R. Baucom, J. S. Baer, J. C. Kircher, D. C. Atkins, The association of therapist empathy and synchrony in vocally encoded arousal. *J. Couns. Psychol.* **61**, 146–153 (2014).

17. F. Ramseyer, W. Tschacher, Nonverbal synchrony in psychotherapy: Coordinated body movement reflects relationship quality and outcome. *J. Consult. Clin. Psychol.* **79**, 284–295 (2011).

18. A. Finset, K. Ørnes, Empathy in the clinician–patient relationship: The role of reciprocal adjustments and processes of synchrony. *J. Patient Exp.* **4**, 64–68 (2017).

19. C. D. Marci, J. Ham, E. Moran, S. P. Orr, Physiologic correlates of perceived therapist empathy and social-emotional process during psychotherapy. *J. Nerv. Ment. Dis.* **195**, 103–111 (2007).

20. M. B. Schippers, A. Roebroeck, R. Renken, L. Nanetti, C. Keysers, Mapping the information flow from one brain to another during gestural communication. *Proc. Natl. Acad. Sci. U.S.A.* **107**, 9388–9393 (2010).

21. L. J. Silbert, C. J. Honey, E. Simony, D. Poeppel, U. Hasson, Coupled neural systems underlie the production and comprehension of naturalistic narrative speech. *Proc. Natl. Acad. Sci. U.S.A.* **111**, E4687–E4696 (2014).

22. G. J. Stephens, L. J. Silbert, U. Hasson, Speaker–listener neural coupling underlies successful communication. *Proc. Natl. Acad. Sci. U.S.A.* **107**, 14425–14430 (2010).

23. E. Bilek, M. Ruf, A. Schäfer, C. Akdeniz, V. D. Calhoun, C. Schmahl, C. Demanuele, H. Tost, P. Kirsch, A. Meyer-Lindenberg, Information flow between interacting human brains: Identification, validation, and relationship to social expertise. *Proc. Natl. Acad. Sci. U.S.A.* **112**, 5207–5212 (2015).

24. S. W. Mercer, M. Maxwell, D. Heaney, G. C. Watt, The Consultation and Relational Empathy (CARE) Measure: Development and preliminary validation and reliability of an empathy-based consultation process measure. *Fam. Pract.* **21**, 699–705 (2004).

25. A. P. Burgess, On the interpretation of synchronization in EEG hyperscanning studies: A cautionary note. *Front. Hum. Neurosci.* **7**, 881 (2013).

26. S. L. Koole, W. Tschacher, Synchrony in psychotherapy: A review and an integrative framework for the therapeutic alliance. *Front. Psychol.* **7**, 862 (2016).

27. D. A. Matthews, A. L. Suchman, W. T. Branch Jr, Making connexions: Enhancing the therapeutic potential of patientclinician relationships. *Ann. Intern. Med.* **118**, 973–977 (1993).

28. M. Schurz, M. G. Tholen, J. Perner, R. B. Mars, J. Sallet, Specifying the brain anatomy underlying temporo-parietal junction activations for theory of mind: A review using probabilistic atlases from different imaging modalities. *Hum. Brain Mapp.* **38**, 4788–4805 (2017).

29. S. G. Shamay-Tsoory, J. Aharon-Peretz, D. Perry, Two systems for empathy: A double dissociation between emotional and cognitive empathy in inferior frontal gyrus versus ventromedial prefrontal lesions. *Brain* **132**, 617–627 (2009).

30. L. Y. Atlas, T. D. Wager, A meta-analysis of brain mechanisms of placebo analgesia: Consistent findings and unanswered questions, in *Placebo*, F. Benedetti, P. Enck, E. Frisaldi, M. Schedlowski, Eds. (Springer Berlin Heidelberg, 2014), vol. 225, pp. 37–69.

31. A. Tinnermann, S. Geuter, C. Sprenger, J. Finsterbusch, C. Büchel, Interactions between brain and spinal cord mediate value effects in nocebo hyperalgesia. *Science* **358**, 105–108 (2017).

32. F. Eippert, U. Bingel, E. D. Schoell, J. Yacubian, R. Klinger, J. Lorenz, C. Büchel, Activation of the opioidergic descending pain control system underlies placebo analgesia. *Neuron* **63**, 533–543 (2009).

33. D.-M. Ellingsen, J. Wessberg, M. Eikemo, J. Liljencrantz, T. Endestad, H. Olausson, S. Leknes, Placebo improves pleasure and pain through opposite modulation of sensory processing. *Proc. Natl. Acad. Sci. U.S.A.* **110**, 17993–17998 (2013).

34. E. Carlino, E. Frisaldi, F. Benedetti, Pain and the context. *Nat. Rev. Rheumatol.* **10**, 348–355 (2014).

35. I. Kirsch, Response expectancy as a determinant of experience and behavior. *Am. Psychol.* **40**, 1189–1202 (1985).

36. K. J. Sherman, D. C. Cherkin, L. Ichikawa, A. L. Avins, K. Delaney, W. E. Barlow, P. S. Khalsa, R. A. Deyo, Treatment expectations and preferences as predictors of outcome of acupuncture for chronic back pain. *Spine* **35**, 1471–1477 (2010).

37. N. E. Foster, E. Thomas, J. C. Hill, E. M. Hay, The relationship between patient and practitioner expectations and preferences and clinical outcomes in a trial of exercise and acupuncture for knee osteoarthritis. *Eur. J. Pain* **14**, 402–409 (2010).

38. K. Jensen, I. Kirsch, S. Odmalm, T. J. Kaptchuk, M. Ingvar, Classical conditioning of analgesic and hyperalgesic pain responses without conscious awareness. *Proc. Natl. Acad. Sci. U.S.A.* **112**, 7863–7867 (2015).

39. T. D. Wager, L. Y. Atlas, M. A. Lindquist, M. Roy, C.-W. Woo, E. Kross, An fMRI-based neurologic signature of physical pain. *N. Engl. J. Med.* **368**, 1388–1397 (2013).

40. C.-W. Woo, L. Schmidt, A. Krishnan, M. Jepma, M. Roy, M. A. Lindquist, L. Y. Atlas, T. D. Wager, Quantifying cerebral contributions to pain beyond nociception. *Nat. Commun.* **8**, 14211 (2017).

41. J. Panksepp, J. B. Panksepp, Toward a cross-species understanding of empathy. *Trends Neurosci.* **36**, 489–496 (2013).

42. L. Koban, A. Ramamoorthy, I. Konvalinka, Why do we fall into sync with others? Interpersonal synchronization and the brain's optimization principle. *Soc. Neurosci.* **14**, 1–9 (2019).

43. L. Beckes, J. A. Coan, Social baseline theory: The role of social proximity in emotion and economy of action. *Soc. Personal. Psychol. Compass.* **5**, 976–988 (2011).

44. H. L. Fields, Understanding how opioids contribute to reward and analgesia. *Reg. Anesth Pain Med.* **32**, 242–246 (2007).

45. L. Steinkopf, The signaling theory of symptoms: An evolutionary explanation of the placebo effect. *Evol. Psychol.* **13**, 10.1177/1474704915600559 (2015).

46. S. P. Lord, E. Sheng, Z. E. Imel, J. Baer, D. C. Atkins, More than reflections: Empathy in motivational interviewing includes language style synchrony between therapist and client. *Behav. Ther.* **46**, 296–303 (2015).

47. W. Tschacher, G. M. Rees, F. Ramseyer, Nonverbal synchrony and affect in dyadic interactions. *Front. Psychol.* **5**, 1323 (2014).

48. M. L. Knapp, J. A. Hall, *Nonverbal Communication in Human Interaction* (Harcourt Brace College, ed. 4, 1997).

49. F. Wolfe, W. Häuser, Fibromyalgia diagnosis and diagnostic criteria. *Ann. Med.* **43**, 495–502 (2011).

50. S. T. A. Yeung, B. Colagiuri, P. F. Lovibond, L. Colloca, Partial reinforcement, extinction, and placebo analgesia. *Pain* **155**, 1110–7 (2014).

51. M. Phillips, A. Lorie, J. Kelley, S. Gray, H. Riess, Long-term effects of empathy training in surgery residents: A one year follow-up study. *Eur J Pers Cent Healthc* **1**, 326–332 (2013).

52. H. Riess, J. M. Kelley, R. Bailey, P. M. Konowitz, S. T. Gray, Improving empathy and relational skills in otolaryngology residents: A pilot study. *Otolaryngol. Head Neck Surg.* **144**, 120–122 (2011).

53. P. Ekman, E. Rosenberg, *What the face reveals: Basic and applied studies of spontaneous expression using the Facial Action Coding System (FACS)* (Oxford University Press, USA, 1997).

54. A. N. Meltzoff, M. K. Moore, Imitation of facial and manual gestures by human neonates. *Science* **198**, 74–78 (1977).

55. H. Rayson, J. J. Bonaiuto, P. F. Ferrari, L. Murray, Early maternal mirroring predicts infant motor system activation during facial expression observation. *Sci. Rep.* **7**, 11738 (2017).

56. T. L. Chartrand, J. A. Bargh, The chameleon effect: The perception–behavior link and social interaction. *J. Pers. Soc. Psychol.* **76**, 893–910 (1999).

57. M. Salazar Kämpf, H. Liebermann, R. Kerschreiter, S. Krause, S. Nestler, S. C. Schmukle, Disentangling the sources of mimicry: Social relations analyses of the link between mimicry and liking. *Psychol. Sci.* **29**, 131–138 (2018).

58. A. Finset, T. A. Mjaaland, The medical consultation viewed as a value chain: A neurobehavioral approach to emotion regulation in doctor–patient interaction. *Patient Educ. Couns.* **74**, 323–330 (2009).

59. M. W. Woolrich, T. E. J. Behrens, C. F. Beckmann, M. Jenkinson, S. M. Smith, Multilevel linear modelling for FMRI group analysis using Bayesian inference. *Neuroimage* **21**, 1732–1747 (2004).

60. M. W. Woolrich, B. D. Ripley, M. Brady, S. M. Smith, Temporal autocorrelation in univariate linear modeling of FMRI data. *Neuroimage* **14**, 1370–1386 (2001).

61. D. Rangaprakash, G.-R. Wu, D. Marinazzo, X. Hu, G. Deshpande, Hemodynamic response function (HRF) variability confounds resting-state fMRI functional connectivity. *Magn. Reson. Med.* **80**, 1697–1713 (2018).

62. D. A. Handwerker, J. M. Ollinger, M. D'Esposito, Variation of BOLD hemodynamic responses across subjects and brain regions and their effects on statistical analyses. *Neuroimage* **21**, 1639–1651 (2004).

63. V. Napadow, R. Dhond, K. Park, J. Kim, N. Makris, K. K. Kwong, R. E. Harris, P. L. Purdon, N. Kettner, K. K. S. Hui, Time-variant fMRI activity in the brainstem and higher structures in response to acupuncture. *Neuroimage* **47**, 289–301 (2009).

64. J. Lee, R. L. Lin, R. G. Garcia, J. Kim, H. Kim, M. L. Loggia, I. Mawla, A. D. Wasan, R. R. Edwards, B. R. Rosen, N. Hadjikhani, V. Napadow, Reduced insula habituation associated with amplification of trigeminal brainstem input in migraine. *Cephalalgia* **37**, 1026–1038 (2016).

65. E. A. R. Losin, C.-W. Woo, A. Krishnan, T. D. Wager, M. Iacoboni, M. Dapretto, Brain and psychological mediators of imitation: Sociocultural versus physical traits. *Cult. Brain.* **3**, 93–111 (2015).

66. C. Becchio, A. Cavallo, C. Begliomini, L. Sartori, G. Feltrin, U. Castiello, Social grasping: From mirroring to mentalizing. *Neuroimage* **61**, 240–248 (2012).

67. L. Budell, P. Jackson, P. Rainville, Brain responses to facial expressions of pain: Emotional or motor mirroring? *Neuroimage* **53**, 355–363 (2010).

68. L. Budell, M. Kunz, P. L. Jackson, P. Rainville, Mirroring pain in the brain: Emotional expression versus motor imitation. *PLOS ONE* **10**, e0107526 (2015).

69. E. Vachon-Presseau, S. E. Berger, T. B. Abdullah, L. Huang, G. A. Cecchi, J. W. Griffith, T. J. Schnitzer, A. V. Apkarian, Brain and psychological determinants of placebo pill response in chronic pain patients. *Nat. Commun.* **9**, 3397 (2018).

70. I. Tracey, Getting the pain you expect: Mechanisms of placebo, nocebo and reappraisal effects in humans. *Nat. Med.* **16**, 1277–1283 (2010).

71. M. Amanzio, F. Benedetti, C. A. Porro, S. Palermo, F. Cauda, Activation likelihood estimation meta-analysis of brain correlates of placebo analgesia in human experimental pain. *Hum. Brain Mapp.* **34**, 738–752 (2013).

72. M. D. Lieberman, J. M. Jarcho, S. Berman, B. D. Naliboff, B. Y. Suyenobu, M. Mandelkern, E. A. Mayer, The neural correlates of placebo effects: A disruption account. *Neuroimage* **22**, 447–455 (2004).

73. D. Tingley, T. Yamamoto, K. Hirose, L. Keele, K. Imai, *mediation: R Package for Causal Mediation Analysis* (UCLA Statistics/American Statistical Association, 2014); https://dspace.mit.edu/handle/1721.1/91154.

74. K. Imai, L. Keele, D. Tingley, A general approach to causal mediation analysis. *Psychol. Methods* **15**, 309–334 (2010).

75. P. Rainville, J. S. Feine, M. C. Bushnell, G. H. Duncan, A psychophysical comparison of sensory and affective responses to four modalities of experimental pain. *Somatosens. Mot. Res.* **9**, 265–277 (1992).

76. M. Curatolo, L. Arendt-Nielsen, S. Petersen-Felix, Central hypersensitivity in chronic pain: Mechanisms and clinical implications. *Phys. Med. Rehabil. Clin. N. Am.* **17**, 287–302 (2006).

77. R. Polianskis, T. Graven-Nielsen, L. Arendt-Nielsen, Spatial and temporal aspects of deep tissue pain assessed by cuff algometry. *Pain* **100**, 19–26 (2002).

78. R. Polianskis, T. Graven-Nielsen, L. Arendt-Nielsen, Pressure-pain function in desensitized and hypersensitized muscle and skin assessed by cuff algometry. *J. Pain* **3**, 28–37 (2002).

79. R. Polianskis, T. Graven-Nielsen, L. Arendt-Nielsen, Modality-specific facilitation and adaptation to painful tonic stimulation in humans. *Eur. J. Pain* **6**, 475–484 (2002).

80. R. Polianskis, T. Graven-Nielsen, L. Arendt-Nielsen, Computer-controlled pneumatic pressure algometry—A new technique for quantitative sensory testing. *Eur. J. Pain* **5**, 267–277 (2001).

81. V. Napadow, R. R. Edwards, C. M. Cahalan, G. Mensing, S. Greenbaum, A. Valovska, A. Li, J. Kim, Y. Maeda, K. Park, A. D. Wasan, Evoked pain analgesia in chronic pelvic pain patients using respiratory-gated auricular vagal afferent nerve stimulation. *Pain Med.* **13**, 777–789 (2012).

82. M. L. Loggia, C. Berna, J. Kim, C. M. Cahalan, R. L. Gollub, A. D. Wasan, R. E. Harris, R. R. Edwards, V. Napadow, Disrupted brain circuitry for pain-related reward/punishment in fibromyalgia. *Arthritis Rheumatol.* **66**, 203–212 (2014).

83. J. Kim, M. L. Loggia, C. M. Cahalan, R. E. Harris, F. Beissner, R. G. Garcia, H. Kim, R. Barbieri, A. D. Wasan, R. R. Edwards, V. Napadow, The somatosensory link in fibromyalgia: Functional connectivity of the primary somatosensory cortex is altered by sustained pain and is associated with clinical/autonomic dysfunction. *Arthritis Rheumatol.* **67**, 1395–405 (2015).

84. D. Lakens, A. M. Scheel, P. M. Isager, Equivalence testing for psychological research: A tutorial. *Adv. Methods Pract. Psychol. Sci.* **1**, 259–269 (2018).

85. M. Jenkinson, P. Bannister, M. Brady, S. Smith, Improved optimization for the robust and accurate linear registration and motion correction of brain images. *Neuroimage* **17**, 825–841 (2002).

86. J. L. R. Andersson, S. Skare, J. Ashburner, How to correct susceptibility distortions in spin-echo echo-planar images: Application to diffusion tensor imaging. *Neuroimage* **20**, 870–888 (2003).

87. S. M. Smith, M. Jenkinson, M. W. Woolrich, C. F. Beckmann, T. E. J. Behrens, H. Johansen-Berg, P. R. Bannister, M. De Luca, I. Drobnjak, D. E. Flitney, R. K. Niazy, J. Saunders, J. Vickers, Y. Y. Zhang, N. De Stefano, J. M. Brady, P. M. Matthews, Advances in functional and structural MR image analysis and implementation as FSL. *Neuroimage* **23**, S208–S219 (2004).

88. S. M. Smith, Fast robust automated brain extraction. *Hum. Brain Mapp.* **17**, 143–155 (2002).

89. D. N. Greve, B. Fischl, Accurate and robust brain image alignment using boundary-based registration. *Neuroimage* **48**, 63–72 (2009).

90. M. Jenkinson, S. Smith, A global optimisation method for robust affine registration of brain images. *Med. Image Anal.* **5**, 143–156 (2001).

91. J. Andersson, M. Jenkinson, S. Smith, “Non-linear registration, aka spatial normalisation” (Technical Report TR07JA2, FMRIB, 2010).

92. M. Zunhammer, U. Bingel, T. D. Wager; Placebo Imaging Consortium, Placebo effects on the neurologic pain signature: A meta-analysis of individual participant functional magnetic resonance imaging data. *JAMA Neurol.* **75**, 1321–1330 (2018).
